# Proteomics and mathematical modeling of longitudinal CSF differentiates fast versus slow ALS progression

**DOI:** 10.1002/acn3.51890

**Published:** 2023-08-30

**Authors:** Lucas Vu, Krystine Garcia‐Mansfield, Antonio Pompeiano, Jiyan An, Victoria David‐Dirgo, Ritin Sharma, Vinisha Venugopal, Harkeerat Halait, Guido Marcucci, Ya‐Huei Kuo, Lisa Uechi, Russell C. Rockne, Patrick Pirrotte, Robert Bowser

**Affiliations:** ^1^ Department of Translational Neuroscience Barrow Neurological Institute Phoenix Arizona 85013 USA; ^2^ Cancer & Cell Biology Division Translational Genomics Research Institute Phoenix Arizona 85004 USA; ^3^ Integrated Mass Spectrometry, City of Hope Comprehensive Cancer Center Duarte California 19050 USA; ^4^ International Clinical Research Center St. Anne's University Hospital Brno Czech Republic; ^5^ Department of Hematologic Malignances Translational Science, Gehr Family Center for Leukemia Research Beckman Research Institute, City of Hope Medical Center Duarte California 91010 USA; ^6^ Department of Computational and Quantitative Medicine Beckman Research Institute, City of Hope Medical Center Duarte California 91010 USA

## Abstract

**Objective:**

Amyotrophic lateral sclerosis (ALS) is a heterogeneous disease with a complex etiology that lacks biomarkers predicting disease progression. The objective of this study was to use longitudinal cerebrospinal fluid (CSF) samples to identify biomarkers that distinguish fast progression (FP) from slow progression (SP) and assess their temporal response.

**Methods:**

We utilized mass spectrometry (MS)‐based proteomics to identify candidate biomarkers using longitudinal CSF from a discovery cohort of SP and FP ALS patients. Immunoassays were used to quantify and validate levels of the top biomarkers. A state‐transition mathematical model was created using the longitudinal MS data that also predicted FP versus SP.

**Results:**

We identified a total of 1148 proteins in the CSF of all ALS patients. Pathway analysis determined enrichment of pathways related to complement and coagulation cascades in FPs and synaptogenesis and glucose metabolism in SPs. Longitudinal analysis revealed a panel of 59 candidate markers that could segregate FP and SP ALS. Based on multivariate analysis, we identified three biomarkers (F12, RBP4, and SERPINA4) as top candidates that segregate ALS based on rate of disease progression. These proteins were validated in the discovery and a separate validation cohort. Our state‐transition model determined that the overall variance of the proteome over time was predictive of the disease progression rate.

**Interpretation:**

We identified pathways and protein biomarkers that distinguish rate of ALS disease progression. A mathematical model of the CSF proteome determined that the change in entropy of the proteome over time was predictive of FP versus SP.

## Introduction

Amyotrophic lateral sclerosis (ALS) is a progressive and eventually fatal neurodegenerative disease.^1^ Clinical manifestation of the disease is variable and can mimic other neurodegenerative diseases in early disease course. In addition to clinical heterogeneity, ALS is also associated with numerous pathogenic mechanisms resulting in a heterogeneous patient population.^2^ Biomarkers that highlight specific disease mechanisms may aid drug development or stratify and/or enrich for a more homogenous subset of patients for clinical trials.

Currently, neurofilament light chain (NfL)^3^, ^4^ and phosphorylated neurofilament heavy chain (pNFH)^5^ are the most promising biomarkers for ALS and have been used as exploratory outcome measures in ALS clinical trials,^6^, ^7^ with treatment response reductions of NfL supportive for the recent FDA approval of QALSODY for ALS patients with SOD1 mutations.^8^ Neurofilaments have been studied extensively in cerebrospinal fluid (CSF) and blood and have demonstrated potential diagnostic and prognostic utility.^9^ Levels of neurofilament proteins in CSF have also been shown to differentiate ALS patients with fast disease progression (FP) from those with slow disease progression (SP), with SP typically exhibiting lower levels of neurofilament in biofluids than FPs.^10^, ^11^, ^12^ While recent studies have explored longitudinal changes of individual biomarker proteins in blood or CSF of ALS patients, the use of unbiased methodologies to further discover longitudinal biomarkers for ALS is lacking. Such information may help identify pathologic mechanisms more specific to clinical subtypes of ALS or exhibit alterations throughout the disease course.

In this study, we performed shotgun proteomics on longitudinal CSF samples collected from a discovery cohort of eleven ALS patients consisting of six FPs and five SPs. We identified a combination of three biomarkers, retinoid binding protein 4 (RBP4), kallistatin (SERPINA4), and coagulation factor XII (F12), as the top candidates that segregate FPs from SPs. These results were validated in a separate patient cohort. Using computational workflows and mathematical models we discovered that the variance of the proteome over time within the individual differentiated FP from SP. Our results identified proteins, pathways, and a novel mathematical model that distinguish FP from SP forms of ALS, highlighting the potential to stratify the ALS population based on CSF proteomics that may be beneficial for downstream drug development.

## Methods

### CSF sample collection

CSF was collected over three or more clinic visits from 22 ALS patients and obtained from the Northeast ALS Consortium (NEALS) Biofluid Repository (Table 1) and separated into discovery and validation cohorts. All ALS subjects were defined by El Escorial criteria by experienced neurologists and provided IRB approved informed consent at the time of enrollment. CSF was collected using methods previously described.^13^ Patients were segregated into FP and SP based on the change over time in their ALS Functional Rating Scale revised (ALSFRS‐R) scores which we defined as their progression rate. FP was defined as those that exhibited a disease progression rate ≥1 unit/month and SP had a disease progression rate <0.5 units/month.

**Table 1:** Patient demographics.

Code	Number of visits	Time elapsed between baseline and last visit (months)	Sex	Age at symptom onset	Age at first visit	Change in ALSFRS‐r/month	Rate of progression	Onset site
Discovery								
S1	4	28	F	43	46	0.32	Slow	Limb
S2	4	24	M	53	57	0.29	Slow	Limb
S3	4	20	F	31	65	0.21	Slow	Bulbar
S4	4	18	M	56	57	0.00	Slow	Limb
S5	3	6	F	34	36	0.28	Slow	Limb
F1	4	13	M	56	57	1.69	Fast	Bulbar
F2	4	12	F	65	67	1.66	Fast	Limb
F3	3	8	M	54	55	1.50	Fast	Limb
F4	5	35	F	59	59	1.00	Fast	Limb
F5	4	21	M	52	53	2.33	Fast	Limb
F6	4	20	M	40	41	2.67	Fast	Limb
Validation								
S6	3	12	F	58	65	0.03	Slow	Limb
S7	3	8	M	38	41	0.07	Slow	Limb
S8	3	11	F	64	66	0.48	Slow	Bulbar
S9	3	8	F	27	43	0.03	Slow	Limb
S10	3	9	M	46	54	0.17	Slow	Limb
S11	3	9	M	63	66	0.12	Slow	Limb
F7	3	8	M	N/A	51	1.50	Fast	Limb
F8	3	7	F	52	53	1.37	Fast	Limb
F9	3	6	M	54	55	2.67	Fast	Limb
F10	3	8	F	58	59	2.34	Fast	Limb
F11	3	8	M	75	77	1.05	Fast	Limb

Patient demographics from both discovery and validation cohorts used in this study. Each patient was characterized as having fast progressing (FP) or slow progressing (SP) ALS based on the rate of change in ALSFRS‐r over time. FP was defined as those that exhibited a rate of change ≥1 unit/month. SP was defined as those that exhibited a rate of change <0.5 unit/month. No significant differences in age at onset (*p* = 0.14), onset site (*p* = 0.99) or sex (*p* = 0.57) were observed in the discovery cohort. No significant differences in age at onset (*p* = 0.61), onset site (p = 0.99), or sex (*p* = 0.99) were observed in the validation cohort. F = female; M = male; N/A – not available.

### CSF sample preparation

One milliliter of each CSF sample was thawed on ice and concentrated using Amicon Ultra (3 kDa molecular weight cutoff) centrifugal filter spin columns (Millipore Sigma, Burlington, MA). Columns were conditioned with HPLC grade water and CSF samples were added and spun at 10,000 × g for 45 min. The columns were subsequently inverted and spun at 1000 × g for 2 min and remaining volume (50 μL) collected in low binding tubes. Protein Separation Buffer A (Millipore Sigma, Burlington, MA) was added up to a volume of 200 μL. Concentrated CSF samples were subsequently added to spin cartridges for depletion of six abundant proteins (Agilent, Santa Clara, CA, Cat #5188‐5230). Prior to addition of the CSF samples, each depletion column was conditioned with Buffer A. CSF samples were then loaded and spun at 100 × g for 2 min. Buffer A was added and spun at 100 × g for 3 min. The eluates were collected and subjected to buffer exchange using the Amicon Ultra (3 kDa molecular weight cut‐off) centrifugal filter spin columns. These columns were conditioned with 50 mM ammonium bicarbonate (Ambic) and spun at 10,000 × *g* for 10 min. The eluate from the depletion steps were added to the columns and spun at 10,000 × g for 45 min. 50 mM Ambic was added and spun at 10,000 × *g* for 45 min. The columns were inverted, spun at 1000 × g for 2 min, and the remaining volume (50 μL) collected in low bind tubes. Rapigest (Waters, Milford, MA) was added to the CSF samples for a final concentration of 0.1% (v/v) to aid with denaturation. Proteins were reduced by incubating with 10 mM dithiothreitol (DTT) for 1 h at 60°C and alkylated with 40 mM iodoacetamide for 30 min at room temperature. Digestion was performed using Trypsin Gold (Promega, Madison, WI) at 1:20 ratio and incubated overnight at 37°C. After digestion, trypsin was inactivated by adding trifluoroacetic acid (TFA) to a final concentration of 0.5% (v/v). Peptides were desalted using a Sep‐PAK C18 96 well plate (Waters) and resuspended in 0.1% formic acid solution.

### Mass spectrometry

Mass spectrometry data were acquired on a Thermo Orbitrap Fusion Lumos mass spectrometer interfaced with a Waters nanoAcquity UPLC system. Peptides were first loaded on a trap column (Waters Symmetry C18, 100 Å, 5 μm, 180 μm × 20 mm) at a flowrate of 7.5 μL/min for 10 min using 99.5% A (Water, 0.1% formic acid) and 0.5% B (Acetonitrile, 0.1% formic acid). Post‐loading, the trap column was brought in‐line with the analytical column (Waters Peptide BEH C18, 130 Å, 1.7 μm, 100 μm × 100 mm) and peptides were eluted over 95 min at a flowrate of 500 nL/min using the following gradient: 3–7% B in 1 min, 7–25% B in 72 min, 25–45% B in 10 min, 45–90% B in 0.5 min, isocratic at 90% B for 1 min followed by return to initial conditions in 0.5 min and column re‐equilibration for 10 min. The mass spectrometer was operated in data dependent mode with the following parameters: Spray voltage of 1800 V, ion transfer tube temperature of 275°C, full scan in Orbitrap over the scan range (m/z) of 400–1500 and a resolution of 120,000. Following parent scan, top most abundant m/z peaks were fragmented via HCD (CE 30%) and detection in ion trap. Only precursors with charge state 2–7 selected for MS/MS, and a dynamic exclusion duration of 60 seconds was employed to prevent resampling of the same precursors. The mass spectrometry proteomics data have been deposited to the ProteomeXchange Consortium via the PRIDE partner repository^14^ with the dataset identifier PXD035026.

### Protein identification and quantification

Raw spectra were searched in MaxQuant v1.5.2.8 against a *Homo sapiens* database (Swissprot/UniProtKB, 2017) using the Label Free Quantitation (LFQ) method with trypsin digestion. Peptides were allowed a maximum of two missed cleavages. N‐term acetylation and methionine oxidation were set as variable modifications, and cysteine carbamidomethyl as a fixed modification. Precursor ion tolerance of 4.5 ppm and ion fragment tolerance of 20 ppm were used for peptide confidence.

### Statistical analysis

Data were background corrected and normalized by variance stabilizing transformation (vsn function in limma R package). Batch effects were adjusted (removeBatchEffect function in limma R package) and differential protein abundance analysis was performed with the *DEP* R package.^15^ Normalized LFQ intensities were used to calculate differential protein abundance between FPs and SPs at the first time point and last time point collected for patients in each group. Proteins that exhibited a fold‐change >1.5 and an adjusted *p‐*value ≤0.0125, as assessed using Mann–Whitney test with a Benjamini‐Hochberg *post hoc* correction, were considered as significantly differentially abundant. Principal components analysis (PCA) was performed using mixOmics.^16^ Enriched pathways differentiating FP and SP were determined using Ingenuity Pathway Analysis (IPA) (QIAGEN Inc., https://www.qiagenbioinformatics.com/products/ingenuity‐pathway‐analysis). For each cross‐sectional analysis, protein fold‐changes and adjusted *p*‐values were input into IPA and mapped against the Human Ingenuity Knowledgebase with default parameters. StringDB analysis was applied to the significant proteins to reveal known protein interaction networks between the candidate markers.^17^ Interactions were filtered for a String confidence score ≥0.7 and no additional interactors were allowed. To determine markers that best segregate FPs versus SPs, significant proteins identified in the cross‐sectional analyses were analyzed using the Multivariate Methods with Unbiased Variable package (*MUVR* v0.0.972, R v3.6.1)^18^ and Random Forest modeling. The *plotMV* and *plotVIP* functions of MUVR were utilized with default parameters to graphically represent the precision and strength of the model. Logistic regression was used to determine the sensitivity, specificity, and area‐under‐curve (AUC) of single markers and combined panels of biomarkers, after bootstrapping 1000 samples with 95% confidence intervals for each specified cut‐off value of the criterion. A generalized linear ROC model was generated using the pROC R package.^19^

Longitudinal analysis of the top candidates from MUVR was performed using random slope, random intercept linear mixed modeling with age at first draw as a covariate. This analysis was performed in R using the package *Lme4* to estimate slopes for each biomarker combined with the package *lmerTest* to assess significance between FPs and SPs (*p*‐value <0.05). To further assess differences between FP and SP, significantly differential baseline abundances of each biomarker were also compared using a Mann Whitney test (*p*‐value <0.05 considered significant), using GraphPad Prism v.9.0. Differences in categorical variables (sex and onset site) between FP and SP were assessed with Fisher's exact test (*p*‐value <0.05). Statically significant differences in average age at first draw and age of onset between FP and SP were assessed by a Mann–Whitney test (*p*‐value <0.05).

To create a state‐transition mathematical model of ALS that distinguishes FP from SP, we first performed a PCA of the longitudinal mass spectrometry data from each participant. The first principal component (PC1) separated FP from SP and revealed the dynamics of proteome change over time during ALS progression. Mutual information was used to identify the top 20 proteins most strongly associated with slow or fast progression, with protein abundance as a continuous variable and slow or fast progression as a discrete variable.^20^ The top 20 proteins ranked by mutual information score from this analysis are shown in Table 3. We next used PC1 plotted over time to construct a model of the proteome variance for each ALS patient and used an Ornstein–Uhlenbeck stochastic differential equation to model ALS progression as reflected in PC1 as described below.

### Immunoassays

Validation was performed on top biomarker candidates using both the discovery cohort and a separate validation cohort (Table 1). Measurements of human retinol binding protein 4 (RBP4) and kallistatin (SERPINA4) were performed using DuoSet ELISA kits (R&D systems; Minneapolis,per MN) following the manufacturer's protocol. Measurements of coagulation factor XII (F12) were performed using human F12 ELISA kit (Abcam; Cambridge, MA) also following the manufacturer's instructions. CSF samples were diluted prior to ELISA measurements at 1:100, 1:1000, and 1:10 for SERPINA4, RBP4, and F12, respectively. Assay precision was assessed by average intra and inter coefficient of variations (CVs). Intra‐CVs were less than 7%, 8%, and 8% for SERPINA4, RBP4, and F12, respectively. Inter‐assay CVs were less than 10%, 10%, and 7% for SERPINA4, RBP4, and F12, respectively. All samples and standards were run in duplicate on each plate. Measurements for CHIT1 and NfL were as previously described.^21^

## Results

### Patient cohorts

Eleven ALS patients were used in the discovery cohort (Table 1). Six patients were classified FPs as demonstrated by the change in ALSFRS‐r/month ≥1 while five were SPs as demonstrated by the change in ALSFRS‐r ≤ 0.5/month. No significant differences in age at onset (*p* = 0.14), onset site (*p* = 0.99), or sex (*p* = 0.57) were observed in this cohort between FPs and SPs. In addition to the discovery cohort, we also obtained a separate validation cohort of eleven ALS patients with five FPs and six SPs (Table 1). No significant differences in age at onset (*p* = 0.61), onset site (*p* = 0.99), or sex (*p* = 0.99) were observed in this second cohort.

### Proteomic analyses

**Figure 1:**
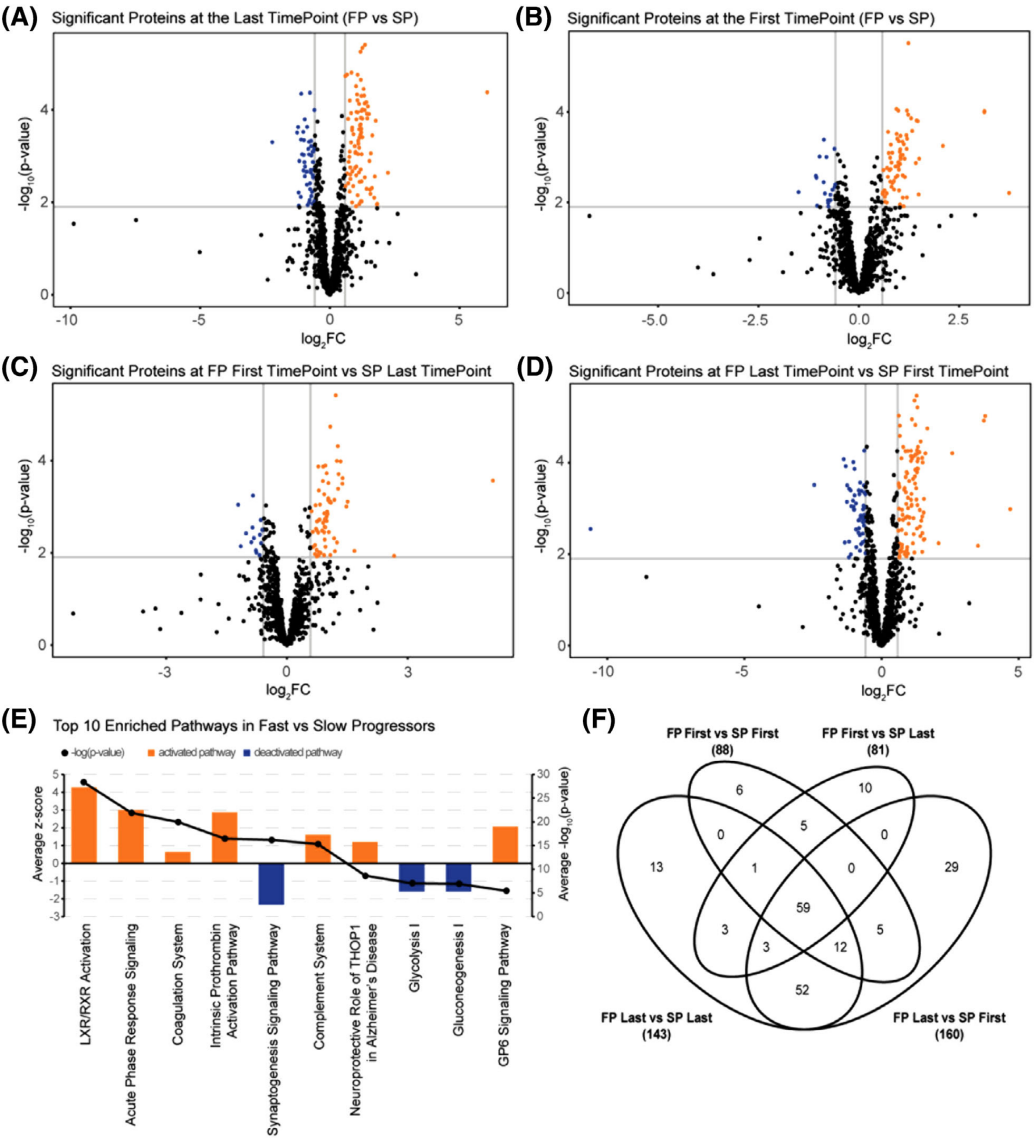
Cross‐sectional pairwise comparisons of the CSF proteome of fast progression (FPs) versus slow progression (SPs). FPs and SPs were compared at (A) the last time point and (B) the first time point. To identify candidate biomarkers that segregate FPs from SPs throughout disease progression, comparisons were made between (C) FPs at the first time point versus SPs at the last time point and conversely (D) FPs at the last time point versus SPs at the first time point. Proteins with a –log_10_ *p* > 1.9 and log_2_ fold change (FC) > 0.58 were considered significant. Differentially abundant proteins are highlighted in orange (increased) and blue (decreased) signifying those that are significant in FPs and SPs, respectively. (E) Bar chart showing top 10 pathways identified by Ingenuity Pathway Analysis on the proteomics data. Orange bars denote upregulated pathways in FPs while blue bars denote downregulated pathways in FPs (i.e., upregulated in SPs). Bars represent the *z*‐score and black line denotes *p*‐values of significant pathways. (F) Venn diagram of all significant proteins from the cross‐sectional comparisons made in A–D.

From the discovery cohort, a total of 1148 proteins were identified across all longitudinal samples from FPs and SPs. To identify candidate biomarkers that were significantly different between FPs and SPs, four cross‐sectional analyses were utilized (Fig. 1). We first compared FPs versus SPs at the first time point (Fig. 1A) and FPs versus SPs at the last time point (Fig. 1B). Second, to verify that the candidate biomarkers could segregate FPs from SPs throughout disease progression, we compared FPs at the first time point versus SPs at the last time point (Fig. 1C) and FPs at the last time point versus SPs at the first time point (Fig. 1D). Significantly enriched pathways in each of the four comparisons were compared to deduce the top pathways that were consistently altered between FPs and SPs (Fig. 1E). Pathways related to inflammatory responses such as *acute phase response signaling*, *coagulation systems*, and *complement systems* were significantly upregulated in the FPs while pathways related to *synaptogenesis* and *glycolysis/gluconeogenesis* were downregulated in FPs suggesting the presence of distinct molecular signatures that contribute to the progression of the disease. Across the four comparisons above, 88, 143, 81, and 160 proteins were considered significantly differentially abundant, respectively. Within this protein list were CHIT1 and NFL, two protein biomarkers previously shown to distinguish between fast and slow disease progression.^12^, ^21^ We note 59 candidates significant across all four group analyses (including CHIT1 but not NEFL) and these were further interrogated (Fig. 1F and Table S1). Within these 59 candidates are 6 members of the serpin family of serine protease inhibitors, 6 members of the apolipoprotein gene family, and 8 members of the complement family. While most of these genes are expressed in the periphery and abundant in the blood, many of them are also expressed in the nervous system.

**Figure 2:**
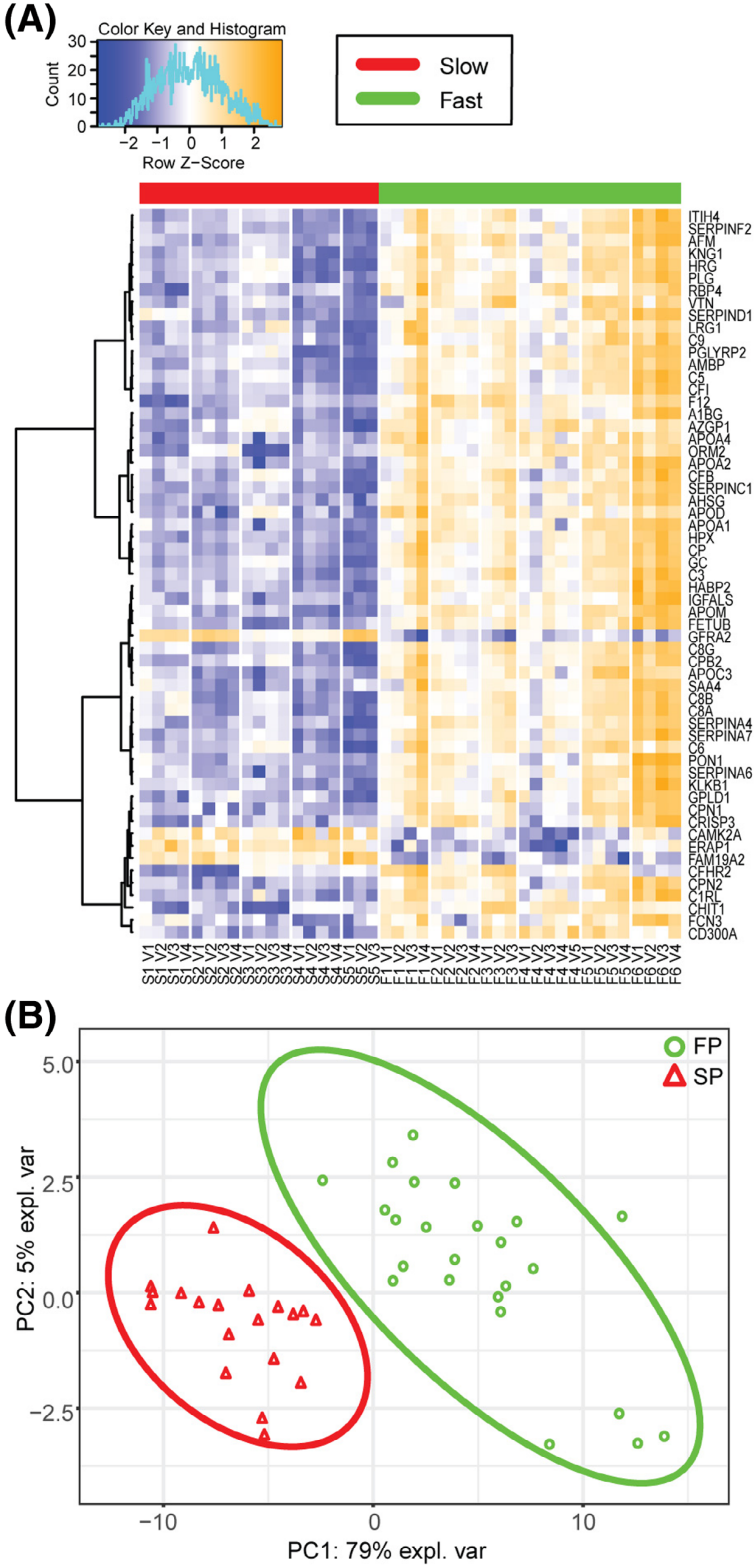
A panel of 59 proteins that segregate FPs and SPs. (A) Supervised clustering of the 59 candidate biomarkers in FPs (red bar) and SPs (green bar). Dendrograms were created using correlation‐based distances and the Ward method of agglomeration was used in the analysis. (B) Principal component analysis based on the panel of 59 proteins reveals a clear segregation between FPs (green circles) and SPs (red triangles).

These 59 candidate biomarkers showed differences in expression between all FP and SP samples across all time points (Fig. 2A). Partial least squares discriminant analysis showed distinct separation of FP and SP (Fig. 2B). Protein–protein interaction analysis using StringDB revealed that 47 out of 59 candidate proteins have annotated associations with each other (Fig. S1). Interestingly, all these proteins with annotated interactions were upregulated in FPs, while the four proteins (GFRA2, CAMK2A, ERAP1, FAM19A2) upregulated in SPs lacked annotated interactions (Fig. S1).

### Multivariate analysis

**Figure 3:**
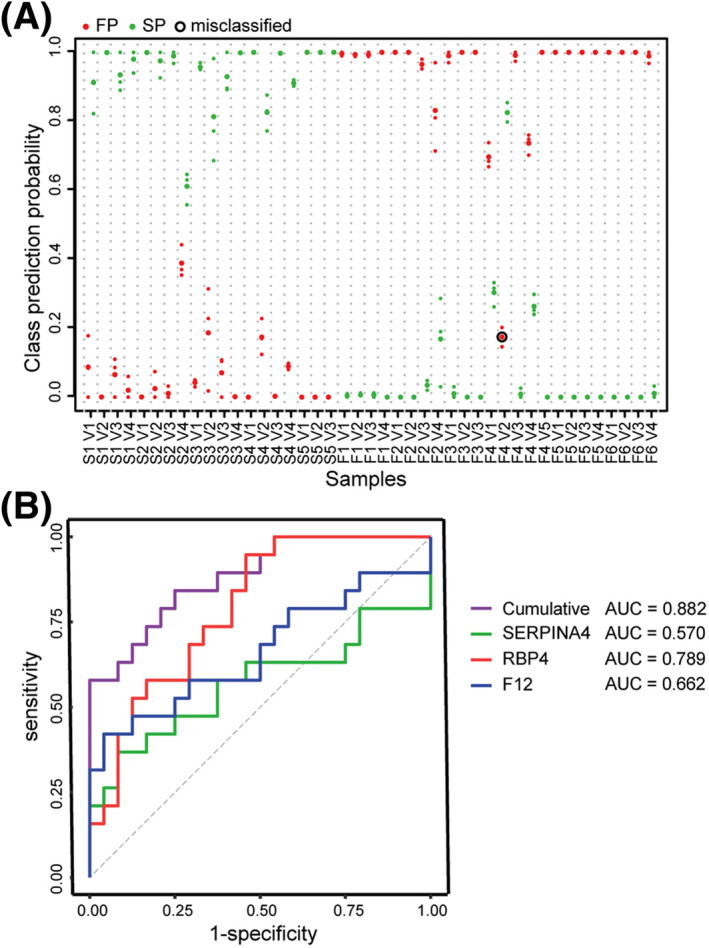
Multivariate analysis was performed using Multivariate Method for Unbiased Variable Selection in R (MUVR) to determine proteins that best distinguish FPs and SPs. (A) Swim lane plot, where each lane shows individual time points and overall predictions for each patient type using the three variables model with FP in red and SP in green. Predictions from individual repetitions are represented by the smaller dots in a lane and the larger dots represent the average class prediction probability across all repetitions. Misclassified samples are highlighted with a black circle (F4 V2). (B) Receiver operator characteristic (ROC) curves using label‐free quantitation (LFQ) intensities from the MS measurements to assess area under the curve (AUC) of SERPINA4 (green), RBP4 (red), F12 (blue), and the combination of all three biomarkers (purple) comparing FP and SP ALS.

Multivariate method for Unbiased Variable Selection in R (MUVR)^18^ was employed to identify optimal candidates that segregate FPs versus SPs. This iterative approach tests combinations of biomarkers and measures their misclassification rates, which is subsequently used to determine the optimal combination to distinguish FPs and SPs. Based on the Random Forest modeling algorithm, it was determined that a three‐biomarker model consisting of Coagulation Factor XII (F12), kallistatin (SERPINA4), and retinol binding protein‐4 (RBP4) best distinguished the two groups. To confirm these results, this model was applied to our discovery cohort to test its accuracy. Three markers accurately classify 43 out of 44 ALS patient samples into FPs and SPs (Fig. 3A). In each lane, the spread in the prediction probabilities demonstrate the precision of the model, with 35 out of 44 samples being correctly classified with a prediction probability >0.9. Only F4 V2 was misclassified as a SP, with a prediction probability of approximately 0.83.

The specificity and sensitivity of each individual biomarker was determined, with AUCs of 0.570, 0.789, and 0.662 for SERPINA4, RBP4, and F12, respectively (Fig. 3B). The combination of all three biomarkers resulted in an improvement over each marker alone (AUC = 0.882). Collectively, these results suggest that the combination of F12, SERPINA4, and RBP4 most clearly distinguish FPs and SPs, as opposed to a single biomarker. Parameters of the logistic regression analysis are provided in Table 2.

**Table 2:** Logistic regression analysis for each individual biomarker and the 3‐biomarker panel.

	AUC (95% CI)	Criterion	Specificity	Sensitivity
Figure 4B				
SERPINA4	0.570 (0.377–0.763)	0.148	0.917	0.368
RBP4	0.789 (0.656–0.923)	0.577	0.542	0.947
F12	0.662 (0.485–0.840)	0.240	0.958	0.421
Panel	0.882 (0.783–0.980)	0.323	0.842	0.750
Figure 6A				
SERPINA4	0.665 (0.493–0.837)	0.181	0.818	0.526
RBP4	0.782 (0.631–0.934)	0.529	0.591	1.00
F12	0.684 (0.518–0.850)	0.229	0.773	0.579
Panel	0.801 (0.654–0.949)	0.376	0.682	1.00
Figure 6B				
SERPINA4	0.904 (0.802–1.00)	0.186	1.00	0.722
RBP4	0.633 (0.440–0.827)	0.576	0.667	0.556
F12	0.715 (0.537–0.892)	0.230	0.933	0.444
Panel	0.907 (0.808–1.00)	0.789	1.00	0.722

**Figure 4:**
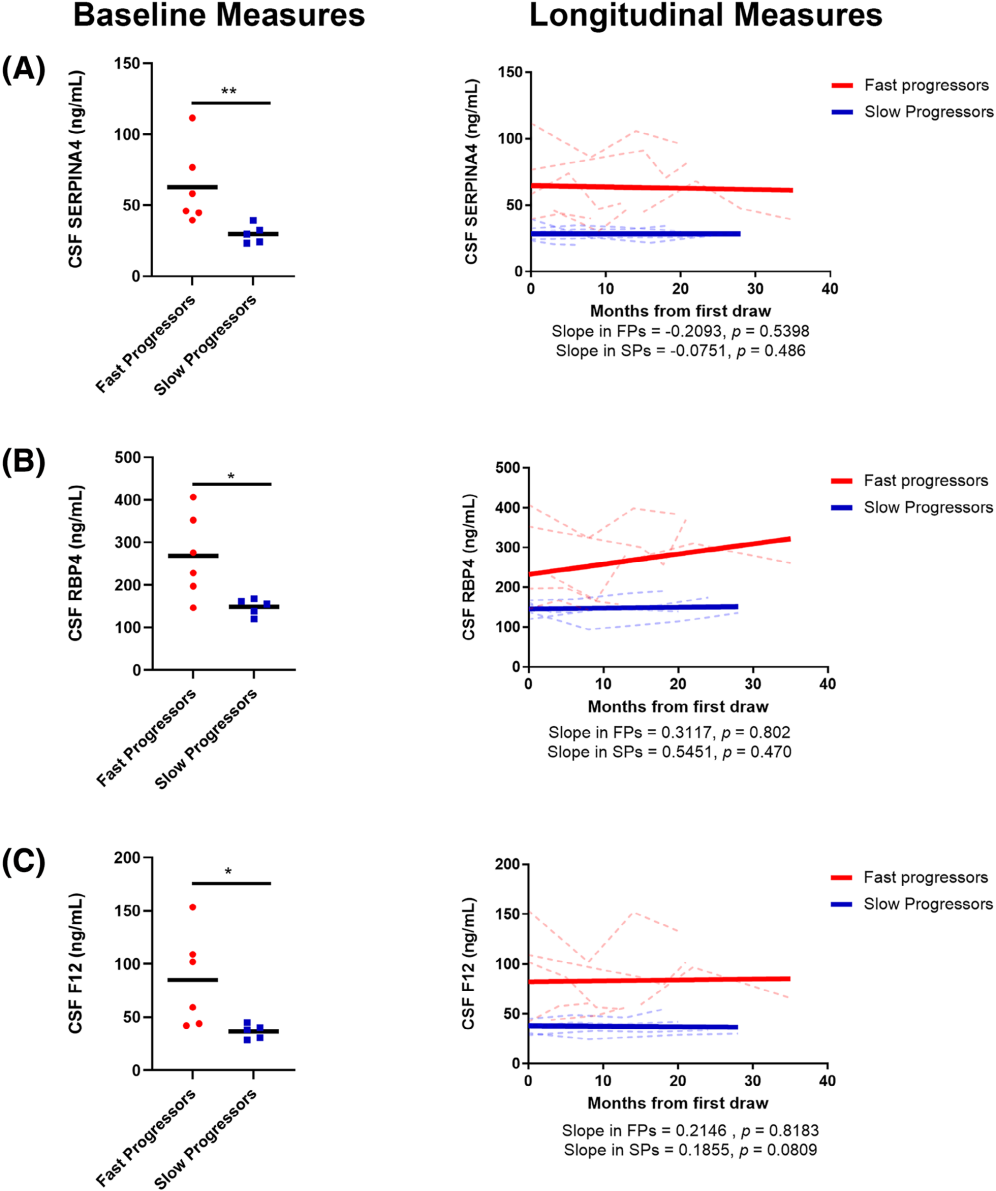
Baseline and longitudinal protein levels of (A) SERPINA4, (B) RBP4 and (C) F12 from ALS patients in the discovery cohort. Solid black bars in the left panels represent the average and each dot represents an individual patient. Each dotted line in the right panels represent an individual patient. The solid lines in the right panels represent the overall linear fit of the longitudinal measurements of each candidate biomarker in FPs (red) and SPs (blue). A Mann–Whitney test was used to assess differences in baseline measurements. **p < 0.01; *p < 0.05. *p*‐values from linear mixed effects modeling indicate the significance level in which the slopes differs from 0 as assessed by r with *p* < 0.05 being considered significant.

Logistic regression analysis for the LC/MS–MS discovery cohort (Fig. 4B), immunoassay results of the discovery cohort (Fig. 7A), and immunoassay results of the validation cohort (Fig. 7B). The area under the curve (AUC) for individual biomarkers and the cumulative 3‐biomarker panel is shown for each experimental condition.

### Validation of RBP4, SERPINA4, and F12

**Figure 5:**
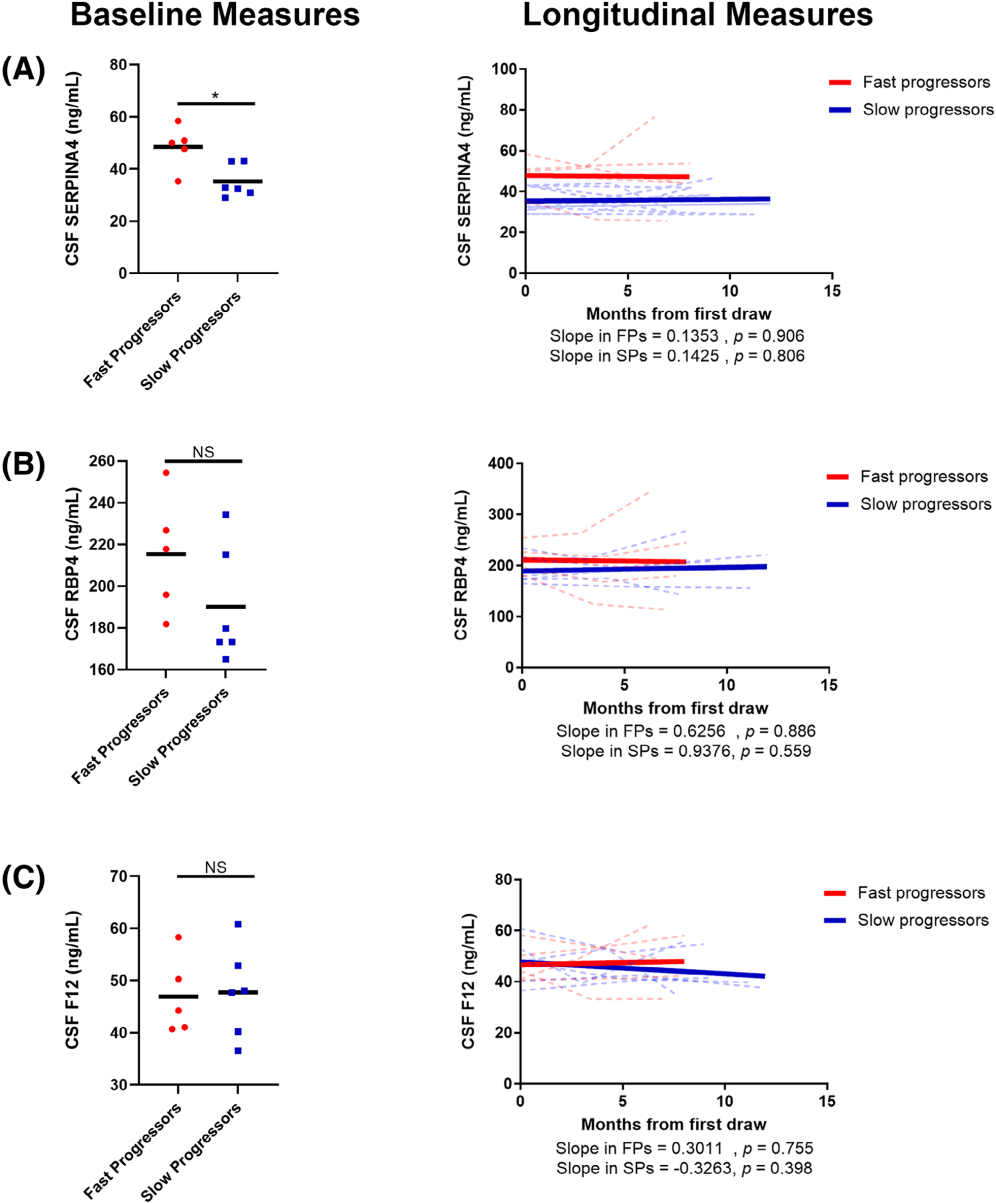
Baseline and longitudinal measures of (A) SERPINA4, (B) RBP4, and (C) F12 from ALS patients in the validation cohort. Solid black bars in the left panels represent the average and each dot represents an individual patient. Each dotted line in the right panels represent an individual patient. The solid lines in the right panels represent the overall linear fit of the longitudinal measurements of each candidate biomarker in FPs (red) and SPs (blue). A Mann–Whitney test was used to assess differences in baseline measurements. *p < 0.05. *p*‐values from linear mixed effects modeling indicate the significance level in which the slopes differs from 0 as assessed by *r* with *p* < 0.05 being considered significant. NS = not significant.

**Figure 6:**
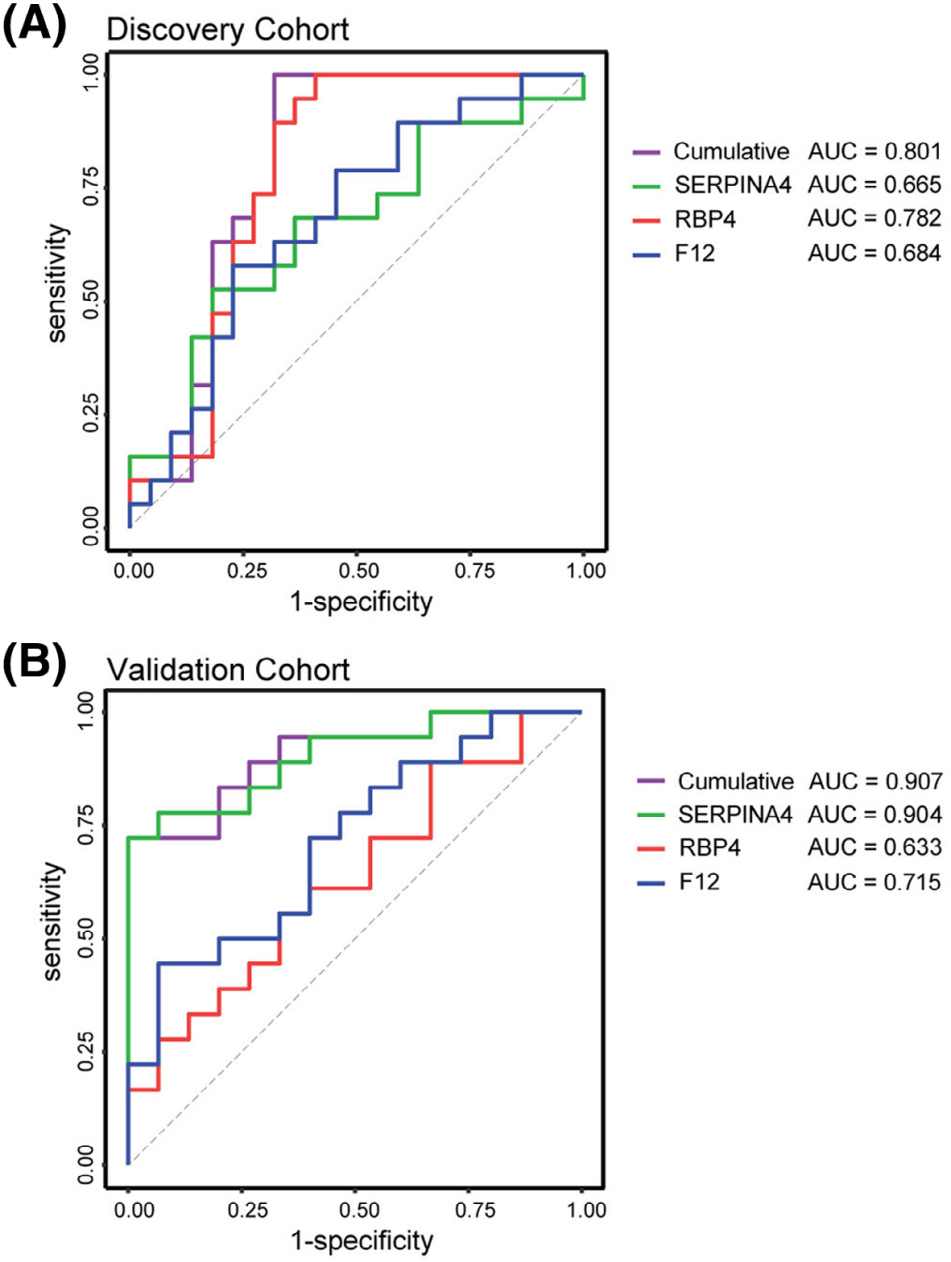
Receiver operator characteristic (ROC) curves using targeted measurements of candidate markers of progression by ELISA in the (A) discovery and (B) validation cohorts. Respective area under the curve (AUC) for SERPINA4 (green), RBP4 (red), F12 (blue), and combination (purple) are provided.

We validated our multivariate analysis using enzyme‐linked immunoassays (ELISA) specific to each protein using CSF from both the discovery and a separate validation cohort (Figs. 4 and 5). In the discovery cohort, baseline levels of all three candidates were significantly higher in FPs (Fig. 4A–C, left). Linear mixed effects modeling analysis revealed that SERPINA4, RBP4, and F12 abundances remain largely unchanged over time in both FP and SP samples (Fig. 4A–C, right). Collectively, these results suggest that, over time, SERPINA4, RBP4, and F12 remain constant but levels are significantly higher in FPs as compared SPs. Within a separate validation cohort, only SERPINA4 exhibited significant differences between FPs and SPs (Fig. 5). To further validate that these biomarkers can distinguish FPs from SPs, ROC analysis was first performed on the discovery cohort, with AUCs of 0.665, 0.782, and 0.684 for SERPINA4, RBP4, and F12, respectively (Fig. 6A). Additionally, the combination of all three biomarkers outperformed (AUC = 0.801) the individual biomarkers. The combination of SERPINA4, F12, and RBP4 was also able to distinguish FPs and SPs (AUC = 0.907) in the validation cohort and outperformed RBP4 (AUC = 0.633) and F12 (AUC = 0.715) alone (Fig. 6B). However, the combination was comparable to SERPINA4 alone (AUC = 0.904) in this separate validation cohort. Parameters of the logistic regression analysis are provided in Table 2. Taken together, these results, which were obtained from two separate cohorts and two methodologies, suggest this biomarker panel distinguishes FPs and SPs.

**Figure 7:**
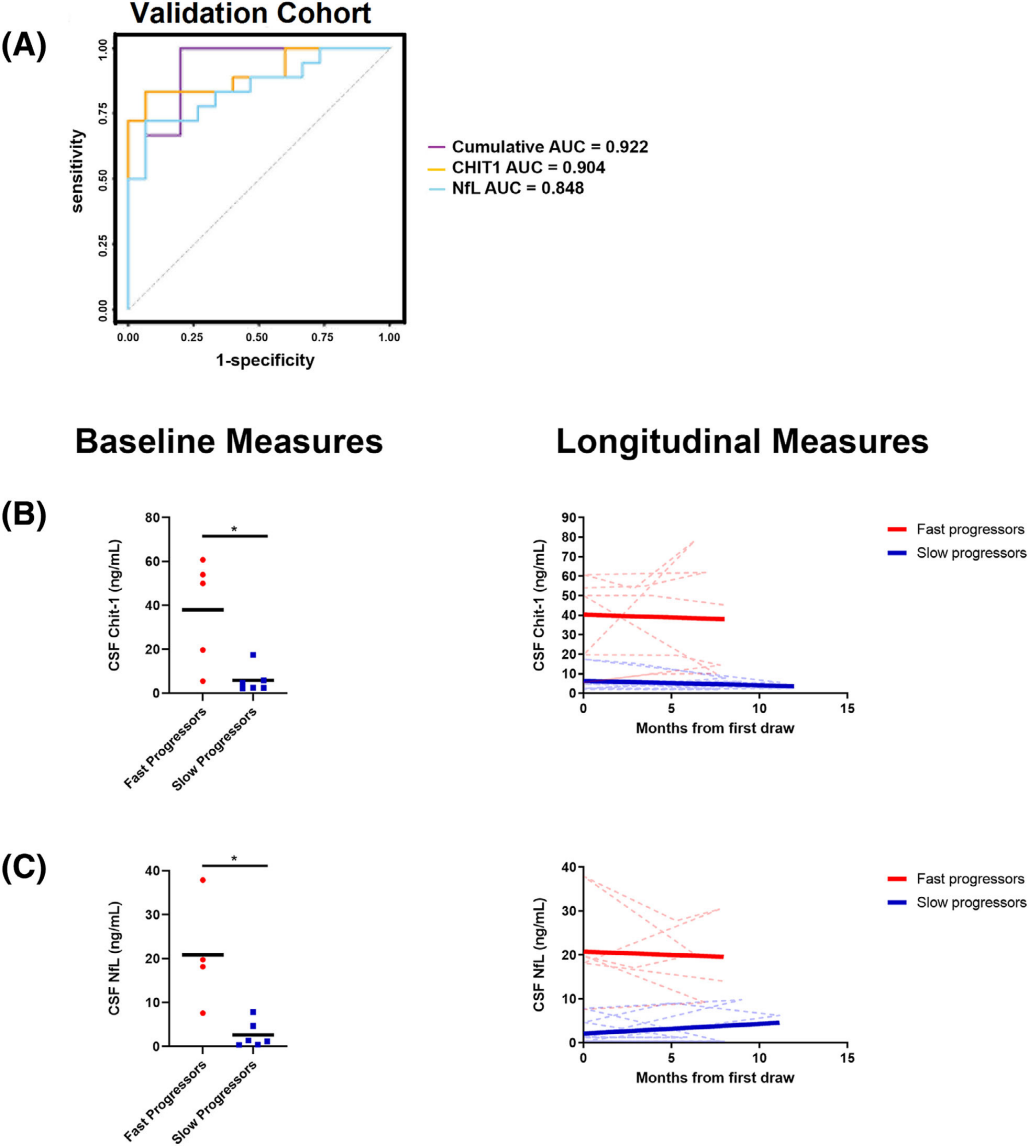
(A) Receiver operator characteristic (ROC) curves using measurements of CHIT1 (orange), NfL (light blue), and combined (purple) in the validation cohort. Baseline and longitudinal measures for (B) CHIT1 and (C) NfL in the validation cohort. For baseline measures, solid black bars in represent the average and each dot represents an individual patient. *p < 0.02. For longitudinal measures the solid lines represent the overall linear fit of the measurements of each protein and the dotted lines represent individual participants.

While NfL, neurofilament medium chain (NfM), and chitinase‐1 (CHIT1) exhibited alterations between fast and slow progressors in the discovery cohort by mass spectrometry (Table S1), NfL and NfM did not exhibit significant changes across all four group comparisons and therefore were not included in the Random Forest multivariate analysis. We independently determined the AUC using NfL and CHIT1 immunoassay data generated from the validation cohort (Fig. 7A). CHIT1 alone distinguishes FP from SP (AUC = 0.904) with NfL measures only providing a small increment (combined AUC = 0.922). Levels of both CHIT1 and NfL protein also exhibited significant baseline differences between SP and FP and remain separated over time in longitudinal samples (Fig. 7B,C).

### Mathematical modeling of ALS disease progression

We next used the longitudinal mass spectrometry data to construct a mathematical model and study the proteome variance as a dynamic biomarker of ALS progression. The model was generated using an Ornstein–Uhlenbeck stochastic differential equation which predicts the steady and irreversible progression of the proteome from a reference state to a subsequent state of ALS.^22^ A similar state‐transition model was recently used to predict the development of acute myeloid leukemia using longitudinal transcriptomics data.^23^ Our state‐transition model describes the rate of change of the proteome over time ($dX_{t}$) as the combination of drift of the proteome away from a reference ALS state (state 1) to a subsequent ALS state (2) (μ) and stochastic fluctuations, which represent proteome entropy over time relative to normal, modeled as a Brownian process ($B_{t}$) with variance $\sqrt{2\beta^{-1}}$ and correlation $\left\langle B_{t_{i}},B_{t_{j}}\right\rangle =\delta_{i,j}$ where $\delta_{i,j}$ is the Dirac Delta function^23^ as follows:

$$\overset{\begin{array}{c} \text{Rate of change}\;\\ \text{of proteome} \end{array}}{\overbrace{dX_{t}}}=\overset{\begin{array}{c} \text{Progression}\\ \text{of}\;\mathrm{ALS} \end{array}}{\overbrace{\theta \left(\mu -X_{t}\right)}}+\overset{\begin{array}{c} \text{Stochastic fluctuations}\\ \left(\text{proteome entropy}\right) \end{array}}{\overbrace{\sqrt{2\beta^{-1}}dB_{t}}}$$

**Figure 8:**
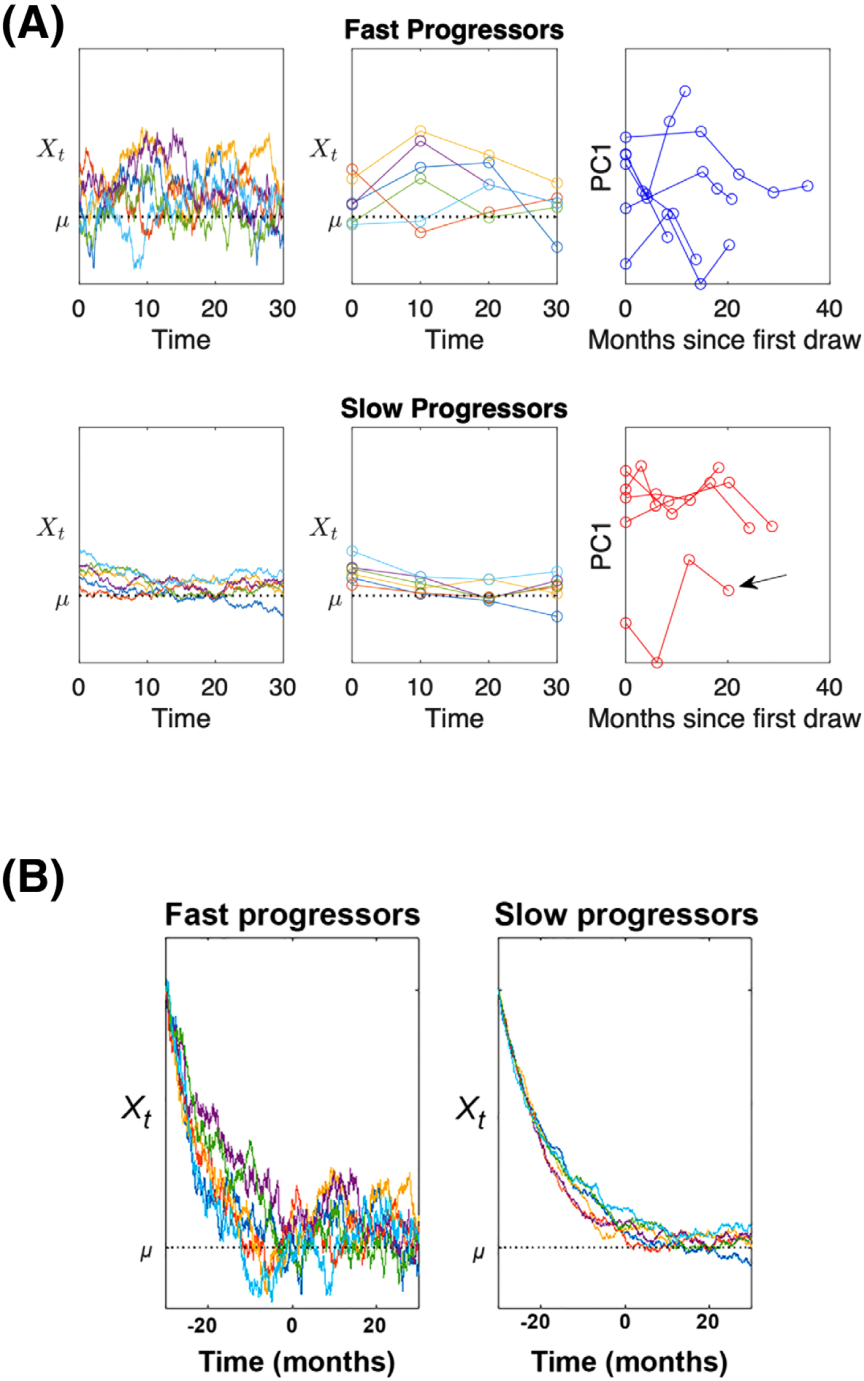
(A) Mathematical model simulations of ALS progression as an Ornstein–Uhlenbeck stochastic process of the state of the proteome ($X_{t}$) in CSF over time (months) for each participant. Each line is a realization of the model based on the mass spectrometry results for each study participant, with the same initial time value (first CSF collection = 0 months). A clinical diagnosis of ALS occurs at a critical threshold corresponding to a state of ALS (μ), which occurs stochastically for each patient (left column). Simulations are down sampled to be comparable to the data (middle column). Patients with fast or slow progression of ALS are modeled as having different rates of ALS ($\theta_{S}<\theta_{F}$) and intrinsic rate of entropy $\beta_{S}^{-1}<\beta_{F}^{-1}$. The first principal component (PC1) plotted over time (right panel) reveals distinct trajectories of fast and slow progressing patients, with proteome changes of slow progressors more consistent over time than fast progressors. One slow progressing patient showed proteome variation more like those of the fast progressors (black arrow). (B) Mathematical model simulations initiated at a presymptomatic state are used to turn back to clock to predict the proteome state prior to the initial sample collection (time = 0 months). The model predicts continued large CSF proteome variance ($X_{t}$) for fast progression when compared to slow progression that would approach time of symptom onset. A clinical diagnosis of ALS would occur once the individual reached a critical threshold corresponding to a clinical state of ALS (μ), which would occur sometime near time *t* = 0 months in this representation.

We applied the mathematical model to mass spectrometry datasets from the FP and SP ALS patient data using the PC1 over time as a representation of the proteome state ($X_{t}$), as PC1 captured the most variation between the fast and slow progressing samples (Fig. 8A). Mutual information was used to identify the top 20 proteins most strongly associated with either SP or FP, with SERPINA4 the top protein (Table 3). All of these proteins except for A2M and KLK6 are also contained in the top 59 protein list in Table S1. The rate of ALS progression is represented as the overall configuration of CSF protein abundances over time and is given by the constant θ, which is proportional to the rate of symptomatic onset. The loss of regulation of the proteome network, through disruption of feedback loops in protein–protein interactions, degenerative processes, etc., is modeled as an increase in overall entropy, or an increase in the number of possible protein abundance configurations (mass peak intensities), or eigenstates, given by the variance of the Brownian process, $\beta^{-1}$. Therefore, to model different rates of ALS progression seen in FP and SP patients, we consider the changes in both the rate constant (θ) which determines the timescale of transition from one state of ALS to another, and the rate of stochastic fluctuation of the proteome over time ($\beta^{-1}$), such that $\theta_{S}<\theta_{F}$, and $\beta_{S}^{-1}<\beta_{F}^{-1}$ (Fig. 8A, left panel). Note the model represents individual patients as distinct colored lines in Figure 8A, with the longitudinal CSF proteome from time 0 (first CSF draw) until 30 months. The overall proteome variance is much greater in FP versus SP patients (Fig. 8A, left panel). The middle panel represents simulations down sampled to be comparable to the data. The right panel is the plot of PC1 values for each FP (blue) and SP (red) over time. While most of the SP patients exhibit a more consistent and stable change over time, one patient displayed more variability and is denoted with an arrow in the lower right panel, with an elevated change in proteome between 10 and 20 months when compared to the state of ALS (μ). This individual may be converting from SP to FP, though additional clinical information to confirm this hypothesis is not available. By initiating the model at a pre‐symptomatic state, we can mathematically turn back the clock to study and predict if or when the proteome may become increasingly unstable prior to symptom onset, when initial CSF samples were collected. These simulations suggest that FP ALS patients may exhibit significant proteome variance very early in the disease process (Fig. 8B). Future studies using longitudinal CSF samples from patients collected before the time of symptom onset will help confirm these results.

**Table 3:** Top 20 ranked proteins used in the mathematical model for ALS disease progression.

Protein	Mutual information score
SERPINA4	0.677
CNTNAP2	0.675
SERPINF2	0.656
HPX	0.641
FETUB	0.634
APOA4	0.604
HABP2	0.602
CP	0.601
KNG1	0.591
SERPINC1	0.580
A2M	0.575
EFNA3	0.571
TCN2	0.565
F12	0.563
C2	0.561
CPN2	0.560
KLK6	0.560
AMBP	0.552
CDH13	0.552
TGFBI	0.547

List of top 20 ranked proteins used in the mathematical model providing mutual information for the mathematical model that distinguishes FP from SP ALS patients.

## Discussion

In this study, we evaluated the CSF proteome in longitudinal samples from FP and SP ALS patients to define proteomic alterations that differentiate FP from SP patients. Overall, a total of 1148 proteins were identified by mass spectrometry across all CSF samples. To interrogate this unbiased longitudinal dataset, we employed multiple cross‐sectional analyses of the CSF proteome at the first and last time points individually and across these two time points (Fig. 1). Combining results from these analyses, we identified 59 biomarkers that can temporally segregate FP and SP ALS (Fig. 2 and Table S1). A multivariate analysis identified a combination of three biomarkers that distinguish FP from SP patients, which were validated in a separate patient cohort (Figs. 3, 4, 5, 6). We also generated a mathematical model that separated FP from SP ALS patients based on the overall proteome variance over time (Fig. 8). High proteome variability corresponds to a fast rate of disease progression, providing an unbiased mass spectrometry approach to predict rate of disease progression.

Pathway analysis determined that proteins related to inflammatory processes such as *acute phase response signaling*, *coagulation systems*, and *complement systems* were upregulated in FPs while pathways related to *synaptogenesis signaling* and *glucose* metabolism were upregulated in SPs (Fig. 1E). A recent study compared the plasma proteome between fast and slow progressors,^24^ with metabolism and glycolysis specifically upregulated in SPs and immune response pathways increased in FPs, similar to our results in the CSF. There was an overlap of specific proteins (APOD, ITIH3, TGFB1, BASP1, CPN1, HBA1, CF1, FCN3, and CPB2) that were significantly altered in FPs versus SPs in both the CSF and plasma proteome. These results highlight common pathways in both the CNS and periphery that differ between FP and SP, though further studies are needed to determine the origin of proteins and relationships to blood–brain barrier (BBB) leakage or change in CSF flow rate.^25^ In a prior study, complement proteins, immunoglobulins, and extracellular matrix proteins exhibited increased abundance over time in ALS CSF whereas proteins related to axonal guidance, synapse assembly, neuropeptide signaling, and RNA processing were reduced over time.^26^ However, this study did not consider rate of disease progression as a variable in their analysis. Our findings provide novel insight by addressing this gap and suggests that distinct molecular mechanisms may drive ALS disease progression in both subgroups. Based on our observation that FPs exhibit increased abundance of many proteins of blood origin including complement and coagulation proteins in CSF, we hypothesize that there may be an increased level of BBB, blood–spinal cord barrier (BSCB), or blood–CSF barrier (BCSFB) disruption in FP ALS. These disruptions have been implicated in ALS pathogenesis based on studies performed in SOD1 mouse models^27^, ^28^, ^29^, ^30^ and in ALS postmortem tissues.^31^, ^32^, ^33^ Recent studies by our group have also demonstrated disruptions in tight junction proteins, compromised vascular integrity, and increased immune cell infiltration into the choroid plexus of ALS cases.^34^ Collectively, these studies highlight BBB, BSCB, and BSCFB dysfunction in ALS pathogenesis and our findings suggest barrier dysfunctions may correlate with disease progression rate. Our unbiased mass spectrometry data also confirmed prior studies indicating chitotriosidase‐1 (CHIT1) and neurofilament proteins (NEFL and NEFM) distinguish fast and slow progressors, which was further validated in a separate patient cohort (Fig. 7).

Additionally, our data demonstrate an enrichment in pathways associated with synaptogenesis and glucose metabolism in SPs. Dysregulation in metabolic pathways have been widely implicated in ALS pathogenesis.^35^ Increased activity of proteins involved in glycolysis (i.e., hexokinase and phosphofructokinase) and the Krebs cycle (i.e., citrate synthase and malate dehydrogenase) has been observed in synaptosomes isolated from the spinal cord and motor cortex of SOD1^G93A^ mice^36^ relative to wild type. Previous metabolomics studies have also demonstrated increased abundances of glycolytic metabolites and intermediates in ALS CSF^37^ and plasma^38^ relative to controls. While the functional consequences of these observations require further exploration, a recent study demonstrated that a high glucose diet elicited a neuroprotective effect by improving motor deficits and survival in Drosophila models that over‐expressed TDP‐43.^39^ Therefore, we propose that alterations in glucose metabolism are occurring in SP ALS patients in an attempt to elicit a neuroprotective response. Further studies are required to explore this possibility and confirm our results.

Our multivariate analysis and Random Forest modeling using the top 59 candidates predicted the combination of three biomarkers (SERPINA4, RBP4, and F12) best distinguish FP and SP ALS across time (Fig. 3). Immunoassays for each protein confirmed the mass spectrometry data in the discovery cohort (Fig. 4) but only SERPINA4 protein was further validated in a separate cohort (Fig. 5). SERPINA4 (Kallistatin) has been identified in a prior proteomic screen of ALS CSF where reduced abundance was observed compared to controls.^40^ Another study demonstrated altered protein levels in the prefrontal cortex of Alzheimer's disease compared to controls.^41^ While these studies highlight differential expression/abundance of SERPINA4, its mechanistic role in ALS and other neurodegenerative diseases is unknown though it does play a role in the inhibition of oxidative stress and inflammation.^42^, ^43^ Therefore, we propose that increased levels of SERPINA4 in CSF of FP ALS patients could indicate a compensatory response to reduce inflammation in the central nervous system. Further studies are needed to address this hypothesis.

We developed a mathematical model that distinguishes FP from SP ALS patients based on the overall CSF proteome variance detected by mass spectrometry. The model identifies significant differences in even the initial CSF sample that can distinguish FP from SP. This novel finding suggests that patients with a rapid disease progression exhibit significant alterations in cellular pathways and secreted proteins, metabolism, and/or involvement of multiple cell types in the disease process that result in considerable protein/peptide variance in the CSF. The overall protein variance is much lower in slow‐progressing ALS patients, consistent with a slow course of disease. The model predicts that overall proteome variance would distinguish fast and slow progressors very early in disease course. While recent ALS clinical trials have focused on one or a small number of biomarkers to examine treatment effect in more rapidly progressing ALS patients,^8^, ^13^, ^44^, ^45^ it may be more beneficial to examine treatment effect on the overall proteome variance as a more unbiased biomarker to demonstrate impact of treatment on overall biologic pathways and cell types linked to disease.

Limitations of our study include the low number of FP and SP patient derived longitudinal samples analyzed by mass spectrometry, and the absence of healthy controls to determine if any proteomic fluctuations over time also occur in an age‐matched control population. An additional limitation to our study is that the time from the first to last sample collection was shorter in the validation cohort when compared to the discovery cohort. Future studies will further explore our biomarker candidates using longitudinal samples from larger numbers of fast and slow progressors to confirm our current findings and extend these findings to asymptomatic mutation carriers.

Taken together, we identified a set of 59 protein biomarkers that best distinguish FP and SP ALS and determined that distinct molecular pathways drive disease progression. Of these 59 proteins, a panel of three biomarkers best distinguished FP from SP ALS. Our novel mathematical model demonstrated that the overall proteome variance differentiates FP from SP ALS patients. These results identify specific protein biomarkers and a model for longitudinal proteome variance that predicts the rate of ALS disease progression as well as pathways that represent potential therapeutic targets and biomarkers for specific clinical subtypes of ALS.

## Author Contributions

L.V., P.P., and R.B. contributed to the conception and design of the study. L.V., J.A., V.D.D, and R.S. contributed to the acquisition of the data and L.V., K.G.M, A.P., R.S., V.V., R.R, P.P., L.U., and R.B. contributed to the analysis of data, with R.R. and L.U. performing the mathematical modeling of the data. L.V., K.G.M, A.P., R.R., P.P., and R.B. contributed to the drafting of the manuscript and figures.

## Conflict of Interest

R.B. is a founder of *n*Vector, Inc., a company developing biomarkers and therapeutics for neurologic disorders. No conflicts of interest were reported by the other authors.

## Supporting information


Figure S1

Table S1

Figure S1 Caption
