# Supplementary material for: Proteomics and mathematical modeling of longitudinal CSF differentiates fast versus slow ALS progression
Source: Ann Clin Transl Neurol. 2023 Aug 30;10(11):2025–42. doi: 10.1002/acn3.51890 (PMC10647001; doi:10.1002/acn3.51890)
Supplement: Supplementary file 2 — Table S1 [file ACN3-10-2025-s001.docx]

**Table S1:** List of proteins identified by the cross-sectional analyses in Fig 1A – D. Proteins with a –log_10_ p > 1.9 and log_2_ fold change (FC) > 0.58 were considered significant. Proteins highlighted in yellow indicate the 59 proteins that overlapped across all four analyses and used in subsequent analyses.

|  | Fast Last v Slow Last | | Fast First v Slow First | | Fast First v Slow Last | | Fast Last v Slow First | |
| --- | --- | --- | --- | --- | --- | --- | --- | --- |
| Gene | pValue | logFC | pValue | logFC | pValue | logFC | pValue | logFC |
| A1BG | ***1.05E-04*** | ***1.22*** | ***4.49E-03*** | ***0.81*** | ***8.67E-03*** | ***0.74*** | ***5.36E-05*** | ***1.29*** |
| ABI3BP | 6.25E-02 | 0.40 | 5.14E-02 | 0.50 | 2.62E-01 | 0.25 | ***1.09E-02*** | ***0.66*** |
| ACAN | 1.51E-01 | 0.67 | 7.06E-02 | 0.74 | 7.43E-01 | -0.12 | ***4.83E-03*** | ***1.54*** |
| ACVR1B | 1.31E-02 | -0.50 | 1.59E-02 | -0.48 | ***3.18E-03*** | ***-0.61*** | 5.86E-02 | -0.37 |
| ADAM10 | 3.05E-01 | -0.16 | ***6.50E-04*** | ***-0.61*** | 1.61E-02 | -0.39 | 2.55E-02 | -0.38 |
| ADO | ***5.01E-04*** | ***-2.22*** | 8.05E-01 | -0.13 | 8.48E-01 | 0.10 | ***3.06E-04*** | ***-2.45*** |
| AFM | ***8.54E-04*** | ***0.98*** | ***1.60E-03*** | ***0.91*** | ***1.39E-03*** | ***0.93*** | ***9.86E-04*** | ***0.97*** |
| AHSG | ***1.74E-05*** | ***1.07*** | ***8.56E-05*** | ***0.94*** | ***1.35E-04*** | ***0.90*** | ***1.12E-05*** | ***1.11*** |
| AMBP | ***7.23E-05*** | ***1.34*** | ***9.87E-04*** | ***1.04*** | ***7.63E-04*** | ***1.07*** | ***9.33E-05*** | ***1.31*** |
| AMY2B | 3.21E-01 | 0.24 | 3.21E-01 | 0.24 | 4.75E-01 | -0.17 | ***1.18E-02*** | ***0.65*** |
| ANG | 2.06E-01 | 0.39 | 1.44E-02 | 0.80 | 2.66E-01 | 0.34 | ***1.01E-02*** | ***0.85*** |
| APCS | 2.63E-02 | 1.18 | ***6.63E-03*** | ***1.49*** | 3.61E-02 | 1.11 | ***4.69E-03*** | ***1.56*** |
| APLP1.1 | ***2.92E-03*** | ***-0.65*** | 1.42E-01 | -0.29 | 9.25E-02 | -0.34 | ***5.07E-03*** | ***-0.60*** |
| APOA1 | ***1.21E-04*** | ***1.43*** | ***2.62E-04*** | ***1.33*** | ***4.09E-04*** | ***1.27*** | ***7.80E-05*** | ***1.48*** |
| APOA2 | ***3.57E-04*** | ***1.63*** | ***1.35E-03*** | ***1.42*** | ***7.77E-04*** | ***1.51*** | ***6.18E-04*** | ***1.54*** |
| APOA4 | ***3.54E-05*** | ***1.30*** | ***9.28E-05*** | ***1.20*** | ***4.88E-05*** | ***1.27*** | ***6.71E-05*** | ***1.23*** |
| APOC2 | ***5.11E-03*** | ***1.41*** | 1.94E-02 | 1.14 | 2.78E-02 | 1.07 | ***3.46E-03*** | ***1.49*** |
| APOC3 | ***6.84E-05*** | ***1.41*** | ***3.88E-04*** | ***1.20*** | ***1.96E-04*** | ***1.28*** | ***1.35E-04*** | ***1.33*** |
| APOD | ***4.65E-05*** | ***1.05*** | ***1.03E-03*** | ***0.78*** | ***2.77E-04*** | ***0.89*** | ***1.70E-04*** | ***0.94*** |
| APOH | ***1.68E-03*** | ***1.02*** | 1.41E-02 | 0.76 | ***1.12E-02*** | ***0.79*** | ***2.15E-03*** | ***0.99*** |
| APOL1 | 1.45E-02 | 0.74 | ***1.40E-03*** | ***1.03*** | ***8.16E-04*** | ***1.09*** | 2.37E-02 | 0.68 |
| APOM | ***1.01E-04*** | ***1.34*** | ***1.37E-04*** | ***1.30*** | ***1.04E-04*** | ***1.33*** | ***1.33E-04*** | ***1.31*** |
| APP | ***2.25E-03*** | ***-0.73*** | 1.13E-01 | -0.35 | 1.46E-01 | -0.32 | ***1.61E-03*** | ***-0.76*** |
| ATP1B1 | 2.05E-01 | 0.20 | ***9.89E-04*** | ***-0.81*** | 3.01E-01 | -0.17 | 3.55E-02 | -0.44 |
| AZGP1 | ***1.11E-04*** | ***1.03*** | ***1.93E-03*** | ***0.77*** | ***3.00E-03*** | ***0.72*** | ***7.12E-05*** | ***1.07*** |
| BAI1 | 6.06E-02 | -0.67 | ***2.84E-03*** | ***-1.06*** | ***9.06E-04*** | ***-1.21*** | 1.42E-01 | -0.51 |
| BASP1 | ***2.34E-04*** | ***-0.88*** | 3.17E-02 | -0.45 | 1.95E-02 | -0.50 | ***4.01E-04*** | ***-0.83*** |
| C1QTNF3 | 2.56E-01 | 0.27 | 3.68E-02 | 0.52 | ***6.80E-03*** | ***0.70*** | 6.99E-01 | 0.09 |
| C1RL | ***7.46E-05*** | ***1.13*** | ***6.27E-03*** | ***0.69*** | ***1.90E-03*** | ***0.81*** | ***2.51E-04*** | ***1.01*** |
| C2 | ***2.75E-04*** | ***0.86*** | ***5.82E-03*** | ***0.60*** | 7.68E-03 | 0.58 | ***2.06E-04*** | ***0.88*** |
| C3 | ***3.38E-04*** | ***0.91*** | ***2.72E-03*** | ***0.72*** | ***2.09E-03*** | ***0.75*** | ***4.42E-04*** | ***0.89*** |
| C4orf48 | 2.61E-02 | -0.53 | 5.44E-02 | -0.45 | 1.97E-01 | -0.30 | ***5.49E-03*** | ***-0.69*** |
| C5 | ***9.13E-04*** | ***1.23*** | ***2.51E-03*** | ***1.09*** | ***3.56E-03*** | ***1.04*** | ***6.40E-04*** | ***1.27*** |
| C6 | ***6.10E-04*** | ***0.96*** | ***3.45E-03*** | ***0.78*** | ***3.62E-03*** | ***0.78*** | ***5.82E-04*** | ***0.96*** |
| C8A | ***6.62E-04*** | ***1.13*** | ***8.86E-04*** | ***1.09*** | ***3.59E-03*** | ***0.92*** | ***1.61E-04*** | ***1.29*** |
| C8B | ***2.61E-04*** | ***1.20*** | ***5.16E-04*** | ***1.12*** | ***1.66E-03*** | ***0.98*** | ***8.15E-05*** | ***1.33*** |
| C8G | ***2.57E-04*** | ***1.21*** | ***3.25E-04*** | ***1.18*** | ***1.44E-03*** | ***1.01*** | ***5.87E-05*** | ***1.38*** |
| C9 | ***1.53E-04*** | ***1.25*** | ***2.78E-03*** | ***0.92*** | ***1.20E-02*** | ***0.75*** | ***3.43E-05*** | ***1.43*** |
| CACNA2D3 | ***2.73E-03*** | ***-0.97*** | 2.00E-02 | -0.72 | 5.37E-02 | -0.58 | ***8.94E-04*** | ***-1.10*** |
| CADM2 | ***9.48E-03*** | ***-0.60*** | 1.43E-01 | -0.32 | 2.88E-01 | -0.23 | ***3.54E-03*** | ***-0.69*** |
| CAMK2A | ***9.18E-04*** | ***-1.07*** | ***3.56E-03*** | ***-0.88*** | ***8.82E-03*** | ***-0.77*** | ***3.73E-04*** | ***-1.18*** |
| CAP1 | ***2.40E-03*** | ***0.67*** | 1.31E-01 | 0.30 | 5.82E-02 | 0.39 | 6.50E-03 | 0.58 |
| CAPG | ***2.43E-04*** | ***0.86*** | ***5.27E-04*** | ***0.80*** | ***1.84E-05*** | ***1.08*** | 7.01E-03 | 0.58 |
| CARTPT | ***5.91E-03*** | ***-0.69*** | 1.67E-02 | -0.58 | 4.96E-02 | -0.47 | ***1.78E-03*** | ***-0.80*** |
| CBLN2 | ***9.21E-04*** | ***-0.80*** | 6.47E-02 | -0.41 | ***4.04E-03*** | ***-0.67*** | 1.75E-02 | -0.54 |
| CBLN4 | ***1.68E-03*** | ***-0.96*** | 1.62E-02 | -0.69 | 2.46E-02 | -0.64 | ***1.07E-03*** | ***-1.01*** |
| CCDC18 | ***5.18E-03*** | ***-0.76*** | 2.03E-02 | -0.61 | 2.70E-02 | -0.58 | ***3.82E-03*** | ***-0.79*** |
| CCL14 | ***1.04E-03*** | ***0.75*** | 3.16E-02 | 0.46 | 7.80E-02 | 0.37 | ***3.57E-04*** | ***0.84*** |
| CD163 | 1.22E-03 | 0.54 | 9.60E-03 | 0.41 | 3.64E-02 | 0.32 | ***2.82E-04*** | ***0.62*** |
| CD300A | ***1.56E-04*** | ***0.71*** | ***2.75E-04*** | ***0.73*** | ***1.35E-04*** | ***0.78*** | ***3.41E-04*** | ***0.65*** |
| CD84 | 7.14E-02 | 0.32 | 9.31E-03 | 0.49 | 3.27E-01 | 0.18 | ***1.01E-03*** | ***0.63*** |
| CDH1 | ***1.27E-03*** | ***1.53*** | 2.09E-01 | 0.51 | 1.78E-02 | 1.01 | 2.06E-02 | 1.02 |
| CDH13 | ***1.34E-03*** | ***-0.66*** | 8.19E-03 | -0.52 | 1.04E-02 | -0.50 | ***1.05E-03*** | ***-0.67*** |
| CDK5RAP2 | ***3.60E-03*** | ***1.14*** | 9.61E-01 | 0.02 | 1.24E-01 | 0.56 | 9.81E-02 | 0.60 |
| CFB | ***1.70E-03*** | ***1.01*** | ***5.18E-03*** | ***0.88*** | ***9.79E-03*** | ***0.80*** | ***8.79E-04*** | ***1.09*** |
| CFH | ***1.33E-03*** | ***0.73*** | ***6.42E-03*** | ***0.59*** | 7.90E-03 | 0.58 | ***1.07E-03*** | ***0.74*** |
| CFHR1 | 1.44E-02 | 0.67 | 2.58E-02 | 0.60 | 4.18E-02 | 0.54 | ***8.60E-03*** | ***0.73*** |
| CFHR2 | ***8.74E-04*** | ***1.58*** | ***1.21E-02*** | ***1.12*** | ***5.93E-03*** | ***1.25*** | ***1.85E-03*** | ***1.45*** |
| CFI | ***4.01E-04*** | ***1.14*** | ***8.98E-04*** | ***1.04*** | ***1.80E-03*** | ***0.96*** | ***1.99E-04*** | ***1.22*** |
| CHGA | ***4.51E-04*** | ***-1.00*** | 1.53E-02 | -0.63 | 1.35E-02 | -0.65 | ***5.17E-04*** | ***-0.98*** |
| CHIT1 | ***4.18E-05*** | ***6.07*** | ***6.17E-03*** | ***3.74*** | ***2.71E-04*** | ***5.12*** | ***1.04E-03*** | ***4.69*** |
| CHL1 | ***6.94E-04*** | ***-0.60*** | 9.42E-03 | -0.43 | 1.42E-02 | -0.40 | ***4.49E-04*** | ***-0.63*** |
| CLMP | 4.61E-01 | 0.26 | 6.87E-01 | 0.13 | ***1.12E-02*** | ***0.92*** | 2.08E-01 | -0.53 |
| CLSTN2 | ***8.92E-03*** | ***-0.78*** | 9.58E-02 | -0.47 | 1.01E-01 | -0.46 | ***8.36E-03*** | ***-0.79*** |
| CNTNAP2 | ***2.18E-03*** | ***-0.91*** | 3.26E-02 | -0.60 | 6.04E-02 | -0.52 | ***1.06E-03*** | ***-0.99*** |
| CP | ***6.21E-05*** | ***1.06*** | ***7.94E-04*** | ***0.83*** | ***8.99E-04*** | ***0.82*** | ***5.49E-05*** | ***1.08*** |
| CPB2 | ***3.09E-04*** | ***1.21*** | ***1.89E-03*** | ***1.00*** | ***2.04E-03*** | ***0.99*** | ***2.85E-04*** | ***1.22*** |
| CPN1 | ***1.72E-04*** | ***1.77*** | ***7.72E-03*** | ***1.20*** | ***9.91E-04*** | ***1.48*** | ***1.51E-03*** | ***1.49*** |
| CPN2 | ***1.52E-04*** | ***1.44*** | ***1.55E-04*** | ***1.44*** | ***2.39E-04*** | ***1.38*** | ***9.86E-05*** | ***1.50*** |
| CRISP3 | ***2.24E-05*** | ***1.19*** | ***1.31E-03*** | ***0.81*** | ***2.01E-03*** | ***0.77*** | ***1.48E-05*** | ***1.23*** |
| DST | ***3.88E-03*** | ***0.71*** | 8.90E-02 | 0.39 | 1.11E-01 | 0.36 | ***2.94E-03*** | ***0.74*** |
| EFNA1 | ***1.07E-02*** | ***-0.66*** | 1.37E-01 | -0.37 | 7.80E-02 | -0.44 | 2.10E-02 | -0.59 |
| EFNA3 | ***7.65E-04*** | ***-0.63*** | 3.71E-03 | -0.52 | 1.79E-03 | -0.57 | 1.60E-03 | -0.58 |
| EFNB3 | 5.81E-02 | -0.41 | 4.53E-02 | -0.44 | 2.15E-01 | -0.26 | ***9.54E-03*** | ***-0.59*** |
| ENPP5 | 1.65E-02 | -0.75 | 2.03E-02 | -0.72 | ***5.87E-03*** | ***-0.88*** | 5.30E-02 | -0.59 |
| EPHA10 | ***6.98E-04*** | ***-0.83*** | 2.54E-01 | -0.21 | ***1.94E-03*** | ***-0.65*** | 6.86E-02 | -0.39 |
| EPHA4 | 6.03E-04 | -0.58 | 7.52E-03 | -0.42 | 9.16E-03 | -0.41 | ***4.90E-04*** | ***-0.59*** |
| EPHB6 | ***9.42E-04*** | ***-1.00*** | 3.79E-02 | -0.58 | 5.04E-02 | -0.54 | ***6.75E-04*** | ***-1.04*** |
| ERAP1 | ***1.99E-03*** | ***-0.73*** | ***4.08E-04*** | ***-0.87*** | ***5.72E-04*** | ***-0.84*** | ***1.42E-03*** | ***-0.76*** |
| F12 | ***5.57E-06*** | ***1.18*** | ***3.01E-06*** | ***1.24*** | ***3.84E-06*** | ***1.21*** | ***4.35E-06*** | ***1.20*** |
| F13B | ***5.82E-03*** | ***0.76*** | 1.31E-02 | 0.67 | ***1.06E-02*** | ***0.70*** | ***7.25E-03*** | ***0.74*** |
| FAM19A1 | 7.18E-02 | -0.59 | ***9.65E-04*** | ***-0.98*** | ***2.82E-03*** | ***-0.85*** | 3.33E-02 | -0.72 |
| FAM19A2 | ***4.39E-04*** | ***-1.11*** | ***9.01E-03*** | ***-0.77*** | ***9.85E-03*** | ***-0.76*** | ***4.00E-04*** | ***-1.13*** |
| FCGBP | ***1.01E-02*** | ***0.86*** | 1.00E-01 | 0.52 | 5.42E-02 | 0.62 | 2.05E-02 | 0.76 |
| FCGR3A | 1.25E-02 | 0.63 | 1.60E-01 | 0.34 | 2.14E-01 | 0.30 | ***8.46E-03*** | ***0.67*** |
| FCN3 | ***9.52E-03*** | ***1.23*** | ***1.08E-03*** | ***1.50*** | ***9.17E-03*** | ***1.19*** | ***1.23E-03*** | ***1.54*** |
| FETUB | ***3.44E-04*** | ***1.18*** | ***1.09E-03*** | ***1.04*** | ***2.72E-03*** | ***0.94*** | ***1.37E-04*** | ***1.28*** |
| FGA | ***4.92E-03*** | ***1.62*** | ***5.61E-04*** | ***2.10*** | 3.84E-02 | 1.14 | ***6.18E-05*** | ***2.58*** |
| FGB | ***1.10E-02*** | ***1.81*** | ***9.53E-05*** | ***3.13*** | 8.74E-02 | 1.16 | ***9.50E-06*** | ***3.78*** |
| FGG | ***1.08E-02*** | ***1.83*** | ***1.02E-04*** | ***3.13*** | 7.48E-02 | 1.22 | ***1.20E-05*** | ***3.73*** |
| FRZB | 1.78E-01 | 0.34 | ***3.36E-04*** | ***1.04*** | 5.25E-02 | 0.50 | ***1.61E-03*** | ***0.88*** |
| FSCN1 | ***1.02E-02*** | ***-0.80*** | 3.56E-01 | -0.27 | 1.12E-01 | -0.47 | 4.64E-02 | -0.60 |
| GAP43 | 5.39E-03 | -0.57 | 2.54E-02 | -0.45 | 7.11E-02 | -0.35 | ***1.66E-03*** | ***-0.67*** |
| GC | ***2.07E-04*** | ***1.19*** | ***1.29E-03*** | ***0.98*** | ***1.88E-03*** | ***0.94*** | ***1.42E-04*** | ***1.23*** |
| GDA | ***9.66E-04*** | ***-0.71*** | 5.96E-02 | -0.37 | 2.73E-02 | -0.44 | ***2.37E-03*** | ***-0.64*** |
| GFRA2 | ***1.60E-04*** | ***-0.97*** | ***5.23E-03*** | ***-0.66*** | ***6.14E-03*** | ***-0.64*** | ***1.36E-04*** | ***-0.98*** |
| GPLD1 | ***2.63E-04*** | ***1.13*** | ***3.68E-03*** | ***0.84*** | ***6.55E-04*** | ***1.03*** | ***1.50E-03*** | ***0.94*** |
| GPNMB | ***2.13E-03*** | ***0.87*** | 5.60E-02 | 0.45 | 1.69E-02 | 0.66 | ***5.91E-03*** | ***0.66*** |
| GPR56 | 7.07E-02 | 0.71 | 1.32E-01 | 0.60 | ***1.11E-02*** | ***1.08*** | 5.42E-01 | 0.23 |
| GRM1 | ***5.07E-04*** | ***0.82*** | 9.87E-03 | 0.56 | 2.85E-02 | 0.47 | ***1.60E-04*** | ***0.91*** |
| HABP2 | ***5.38E-04*** | ***1.10*** | ***1.53E-03*** | ***0.98*** | ***1.28E-03*** | ***1.00*** | ***6.47E-04*** | ***1.08*** |
| HBA1 | 2.43E-02 | -7.47 | 1.98E-02 | -6.71 | 1.87E-01 | -3.57 | ***2.82E-03*** | ***-10.61*** |
| HGFAC | 1.17E-01 | 0.55 | ***2.74E-03*** | ***1.15*** | 4.04E-02 | 0.74 | ***9.67E-03*** | ***0.96*** |
| HPX | ***4.58E-06*** | ***1.25*** | ***9.31E-05*** | ***0.98*** | ***1.28E-04*** | ***0.96*** | ***3.40E-06*** | ***1.28*** |
| HRG | ***9.05E-04*** | ***1.09*** | ***2.56E-03*** | ***0.96*** | ***2.78E-03*** | ***0.95*** | ***8.35E-04*** | ***1.10*** |
| HS1BP3 | ***2.90E-03*** | ***1.35*** | 8.43E-02 | 0.88 | 1.57E-02 | 1.07 | 2.51E-02 | 1.16 |
| HS6ST3 | ***7.64E-03*** | ***-1.12*** | 1.18E-01 | -0.62 | 1.56E-01 | -0.56 | ***5.34E-03*** | ***-1.18*** |
| HSPA8 | 2.60E-02 | -0.42 | 3.95E-02 | -0.38 | 3.13E-01 | -0.18 | ***1.89E-03*** | ***-0.62*** |
| ICAM5 | ***7.33E-03*** | ***-0.71*** | 7.20E-02 | -0.46 | 1.59E-01 | -0.35 | ***2.71E-03*** | ***-0.82*** |
| IGF2 | 6.07E-04 | 0.50 | 3.84E-03 | 0.40 | 6.98E-02 | 0.24 | ***2.59E-05*** | ***0.67*** |
| IGFALS | ***4.68E-04*** | ***1.09*** | ***3.37E-03*** | ***0.87*** | ***3.28E-03*** | ***0.88*** | ***4.81E-04*** | ***1.09*** |
| IGFBP6 | ***1.84E-05*** | ***0.60*** | 1.61E-03 | 0.39 | 3.24E-03 | 0.36 | ***9.34E-06*** | ***0.63*** |
| IGHG3 | ***6.56E-03*** | ***1.57*** | 5.89E-02 | 0.94 | 4.29E-02 | 1.02 | ***8.93E-03*** | ***1.48*** |
| ISLR2 | ***1.27E-03*** | ***-1.18*** | 2.06E-02 | -0.77 | 3.60E-02 | -0.68 | ***7.18E-04*** | ***-1.26*** |
| ITIH1 | ***9.48E-03*** | ***1.19*** | 1.97E-02 | 1.05 | 2.02E-02 | 1.05 | ***9.22E-03*** | ***1.20*** |
| ITIH2 | ***1.08E-02*** | ***1.14*** | 2.44E-02 | 0.98 | 2.11E-02 | 1.01 | 1.26E-02 | 1.11 |
| ITIH3 | ***4.46E-03*** | ***0.90*** | 3.13E-02 | 0.65 | 7.63E-02 | 0.53 | ***1.58E-03*** | ***1.03*** |
| ITIH4 | ***1.49E-04*** | ***1.20*** | ***6.68E-04*** | ***1.03*** | ***2.22E-03*** | ***0.90*** | ***4.52E-05*** | ***1.33*** |
| KIF4A | ***7.98E-03*** | ***1.71*** | 2.98E-01 | 0.62 | ***9.03E-03*** | ***1.68*** | 2.75E-01 | 0.65 |
| KLKB1 | ***1.80E-03*** | ***0.97*** | ***1.59E-03*** | ***0.98*** | ***7.91E-04*** | ***1.06*** | ***3.58E-03*** | ***0.89*** |
| KNG1 | ***4.92E-05*** | ***1.32*** | ***1.15E-04*** | ***1.23*** | ***1.02E-04*** | ***1.24*** | ***5.54E-05*** | ***1.31*** |
| KRT2 | 1.12E-01 | -0.64 | 4.61E-02 | -0.81 | ***7.18E-03*** | ***-1.15*** | 4.32E-01 | -0.31 |
| LBP | ***2.76E-03*** | ***1.73*** | 3.17E-01 | 0.62 | 2.10E-02 | 1.26 | 8.73E-02 | 1.10 |
| LCAT | 1.16E-02 | 0.45 | 3.18E-03 | 0.55 | ***1.24E-03*** | ***0.61*** | 2.75E-02 | 0.39 |
| LCN2 | ***8.08E-04*** | ***0.98*** | 4.43E-01 | 0.20 | 1.76E-02 | 0.65 | 4.59E-02 | 0.53 |
| LCP1 | ***3.11E-03*** | ***0.79*** | 3.68E-01 | 0.22 | 4.77E-02 | 0.50 | 4.25E-02 | 0.51 |
| LINGO1 | ***4.15E-03*** | ***-0.89*** | 2.73E-01 | -0.31 | 3.45E-01 | -0.27 | ***2.87E-03*** | ***-0.93*** |
| LRG1 | ***8.41E-05*** | ***1.47*** | ***1.08E-03*** | ***1.14*** | ***5.17E-03*** | ***0.94*** | ***1.78E-05*** | ***1.67*** |
| LRP1B | 3.61E-02 | 0.71 | 5.41E-01 | 0.15 | 8.14E-01 | -0.06 | ***1.12E-02*** | ***0.91*** |
| LUM | 7.80E-03 | 0.54 | 2.18E-02 | 0.45 | 5.08E-02 | 0.38 | ***3.06E-03*** | ***0.61*** |
| LY6H | ***3.89E-03*** | ***-0.76*** | ***6.73E-03*** | ***-0.71*** | 1.40E-02 | -0.63 | ***1.82E-03*** | ***-0.84*** |
| LYZ | ***2.50E-03*** | ***0.79*** | ***9.60E-03*** | ***0.65*** | 2.77E-02 | 0.54 | ***7.95E-04*** | ***0.90*** |
| MDGA2 | 1.81E-01 | -0.24 | ***4.51E-03*** | ***-0.60*** | 1.47E-02 | -0.53 | 6.54E-02 | -0.31 |
| MDH2 | 5.56E-01 | -0.22 | ***1.18E-02*** | ***-1.04*** | 2.11E-01 | -0.47 | 4.67E-02 | -0.78 |
| MIA | ***2.61E-03*** | ***1.01*** | 2.41E-02 | 0.76 | ***1.00E-02*** | ***0.84*** | ***7.04E-03*** | ***0.93*** |
| MST1 | 1.81E-02 | 0.77 | 2.68E-02 | 0.71 | 5.07E-02 | 0.62 | ***9.12E-03*** | ***0.86*** |
| NEFL | ***2.30E-03*** | ***2.24*** | 4.96E-02 | 1.35 | 2.32E-02 | 1.51 | ***5.74E-03*** | ***2.08*** |
| NEFM | 1.78E-02 | 2.61 | 1.90E-02 | 2.90 | 5.76E-02 | 1.99 | ***6.57E-03*** | ***3.52*** |
| NELL2 | ***1.93E-03*** | ***-0.60*** | 4.40E-02 | -0.36 | 8.55E-02 | -0.31 | ***8.70E-04*** | ***-0.66*** |
| NGFR | ***1.57E-05*** | ***0.83*** | 5.44E-03 | 0.46 | 1.05E-03 | 0.57 | ***7.98E-05*** | ***0.73*** |
| NOXA1 | ***5.29E-03*** | ***1.55*** | 6.92E-02 | 0.73 | 7.85E-02 | 0.88 | ***2.48E-03*** | ***1.40*** |
| NPTX2 | ***1.19E-02*** | ***-1.20*** | 1.99E-01 | -0.58 | 3.33E-01 | -0.43 | ***5.54E-03*** | ***-1.34*** |
| NPTXR | ***6.07E-04*** | ***-0.72*** | 1.08E-02 | -0.50 | 2.27E-02 | -0.44 | ***2.71E-04*** | ***-0.78*** |
| NPY | ***2.16E-03*** | ***-0.89*** | 1.28E-01 | -0.40 | 9.00E-02 | -0.45 | ***3.40E-03*** | ***-0.84*** |
| OPCML | 5.62E-04 | -0.57 | 8.13E-03 | -0.41 | 1.34E-02 | -0.38 | ***3.32E-04*** | ***-0.61*** |
| ORM1 | ***1.22E-02*** | ***1.01*** | ***1.10E-02*** | ***1.03*** | 9.61E-02 | 0.64 | ***1.08E-03*** | ***1.39*** |
| ORM2 | ***1.23E-04*** | ***1.51*** | ***1.60E-04*** | ***1.47*** | ***3.17E-04*** | ***1.38*** | ***6.22E-05*** | ***1.60*** |
| PARD3B | 3.84E-02 | 1.14 | 4.73E-01 | 0.32 | 9.42E-01 | 0.04 | ***4.04E-03*** | ***1.42*** |
| PCDH7 | ***1.14E-02*** | ***-0.85*** | 1.45E-01 | -0.47 | 2.08E-01 | -0.40 | ***7.02E-03*** | ***-0.92*** |
| PCSK1 | ***2.36E-04*** | ***-1.23*** | ***1.22E-02*** | ***-0.76*** | 2.30E-02 | -0.68 | ***1.19E-04*** | ***-1.31*** |
| PCSK2 | ***2.05E-03*** | ***-1.02*** | ***6.32E-03*** | ***-0.85*** | 3.92E-02 | -0.61 | ***3.11E-04*** | ***-1.25*** |
| PDGFB | 1.06E-02 | -0.51 | ***3.99E-03*** | ***-0.59*** | 5.00E-03 | -0.57 | 8.51E-03 | -0.53 |
| PGLYRP2 | ***1.60E-04*** | ***1.02*** | ***1.83E-03*** | ***0.79*** | ***3.19E-03*** | ***0.74*** | ***9.17E-05*** | ***1.07*** |
| PIANP | 1.82E-02 | -0.62 | 1.78E-02 | -0.63 | 5.05E-02 | -0.50 | ***6.02E-03*** | ***-0.74*** |
| PLG | ***2.16E-04*** | ***1.16*** | ***7.47E-04*** | ***1.02*** | ***7.15E-04*** | ***1.03*** | ***2.26E-04*** | ***1.15*** |
| PNOC | ***3.11E-04*** | ***-1.26*** | ***1.02E-02*** | ***-0.76*** | 3.13E-02 | -0.65 | ***8.39E-05*** | ***-1.38*** |
| PON1 | ***3.53E-03*** | ***1.22*** | ***7.81E-03*** | ***1.09*** | ***4.81E-03*** | ***1.17*** | ***5.76E-03*** | ***1.14*** |
| PPP5C | 5.03E-01 | -0.22 | ***5.87E-03*** | ***-1.50*** | 3.85E-02 | -1.02 | 4.23E-02 | -0.70 |
| PTPRN2 | ***4.70E-04*** | ***-0.72*** | 1.07E-02 | -0.49 | 1.21E-02 | -0.48 | ***4.11E-04*** | ***-0.73*** |
| QPCT | ***1.35E-03*** | ***-0.64*** | 4.68E-03 | -0.52 | 4.53E-03 | -0.52 | ***1.39E-03*** | ***-0.64*** |
| QSOX2 | 1.64E-02 | 0.48 | 7.54E-02 | 0.34 | ***9.30E-04*** | ***0.71*** | 5.39E-01 | 0.11 |
| RBP4 | ***3.07E-05*** | ***1.01*** | ***8.06E-04*** | ***0.74*** | ***4.42E-04*** | ***0.79*** | ***5.54E-05*** | ***0.96*** |
| RNASE4 | 1.81E-02 | 0.53 | ***5.41E-03*** | ***0.64*** | 6.14E-02 | 0.41 | ***1.38E-03*** | ***0.76*** |
| RTN4RL1 | 8.55E-02 | -0.46 | ***8.84E-03*** | ***-0.70*** | ***1.12E-02*** | ***-0.68*** | 7.04E-02 | -0.48 |
| SAA4 | ***2.25E-03*** | ***1.12*** | ***5.41E-03*** | ***1.00*** | ***7.37E-03*** | ***0.96*** | ***1.63E-03*** | ***1.17*** |
| SCG2 | ***2.60E-03*** | ***-0.66*** | 3.23E-02 | -0.44 | 4.09E-02 | -0.42 | ***1.99E-03*** | ***-0.68*** |
| SECTM1 | 1.04E-01 | 0.54 | 3.82E-02 | 0.67 | 3.61E-01 | 0.28 | ***8.07E-03*** | ***0.92*** |
| SELL | ***4.49E-03*** | ***0.60*** | 4.20E-02 | 0.42 | 2.10E-02 | 0.48 | 9.75E-03 | 0.53 |
| SEPP1 | 7.20E-03 | 0.43 | 2.65E-03 | 0.50 | 5.10E-02 | 0.30 | ***3.09E-04*** | ***0.63*** |
| SERPINA10 | 2.21E-02 | 0.94 | 5.76E-02 | 0.73 | 3.10E-01 | 0.38 | ***2.75E-03*** | ***1.29*** |
| SERPINA3 | ***1.94E-03*** | ***1.19*** | 1.32E-02 | 0.91 | 2.83E-02 | 0.79 | ***8.45E-04*** | ***1.31*** |
| SERPINA4 | ***4.99E-05*** | ***1.22*** | ***4.41E-04*** | ***0.99*** | ***3.06E-04*** | ***1.03*** | ***7.14E-05*** | ***1.18*** |
| SERPINA5 | ***7.02E-03*** | ***1.07*** | 2.40E-02 | 0.87 | 4.35E-02 | 0.77 | ***3.64E-03*** | ***1.17*** |
| SERPINA6 | ***3.93E-04*** | ***1.25*** | ***6.53E-04*** | ***1.19*** | ***7.10E-04*** | ***1.18*** | ***3.61E-04*** | ***1.26*** |
| SERPINA7 | ***1.13E-04*** | ***1.08*** | ***3.89E-04*** | ***0.96*** | ***5.37E-04*** | ***0.93*** | ***8.19E-05*** | ***1.11*** |
| SERPINC1 | ***3.92E-06*** | ***1.35*** | ***3.05E-04*** | ***0.94*** | ***1.88E-04*** | ***0.99*** | ***6.17E-06*** | ***1.31*** |
| SERPIND1 | ***1.76E-03*** | ***0.79*** | ***7.48E-03*** | ***0.65*** | ***7.43E-03*** | ***0.65*** | ***1.77E-03*** | ***0.78*** |
| SERPINF2 | ***9.82E-05*** | ***1.26*** | ***4.54E-04*** | ***1.09*** | ***7.16E-04*** | ***1.04*** | ***6.26E-05*** | ***1.31*** |
| SERPING1 | ***1.17E-04*** | ***0.82*** | ***1.92E-03*** | ***0.61*** | 4.01E-03 | 0.56 | ***5.59E-05*** | ***0.87*** |
| SERPINI1 | ***1.01E-04*** | ***-0.60*** | 1.20E-03 | -0.47 | 2.23E-03 | -0.43 | ***5.44E-05*** | ***-0.63*** |
| SEZ6L | 2.06E-03 | -0.57 | 1.83E-02 | -0.42 | 3.72E-02 | -0.36 | ***9.32E-04*** | ***-0.63*** |
| SLITRK1 | ***4.93E-04*** | ***-0.81*** | 7.08E-03 | -0.58 | 7.12E-03 | -0.58 | ***4.89E-04*** | ***-0.81*** |
| SLITRK5 | 3.49E-01 | -0.35 | ***2.58E-03*** | ***-1.07*** | ***3.76E-03*** | ***-1.02*** | 2.81E-01 | -0.40 |
| SNED1 | 5.91E-02 | 0.47 | 1.33E-01 | 0.37 | 5.06E-01 | 0.16 | ***9.00E-03*** | ***0.68*** |
| SOD3 | 2.38E-02 | 0.60 | ***1.00E-02*** | ***0.69*** | 3.70E-02 | 0.55 | ***6.24E-03*** | ***0.75*** |
| SST | ***6.15E-03*** | ***-1.20*** | 2.54E-01 | -0.46 | 1.79E-01 | -0.55 | ***1.01E-02*** | ***-1.12*** |
| TCN2 | ***6.30E-05*** | ***0.77*** | 2.58E-03 | 0.53 | 3.59E-03 | 0.50 | ***4.53E-05*** | ***0.79*** |
| TF | 7.26E-02 | 1.81 | 1.97E-02 | 2.30 | ***1.16E-02*** | ***2.66*** | 1.25E-01 | 1.45 |
| TGFBI | ***1.73E-05*** | ***0.66*** | 1.02E-03 | 0.46 | 1.15E-03 | 0.45 | ***1.54E-05*** | ***0.67*** |
| TIMP1 | 5.44E-03 | 0.56 | 3.54E-02 | 0.41 | 9.25E-02 | 0.32 | ***1.75E-03*** | ***0.65*** |
| TMEM132B | ***4.46E-05*** | ***-1.11*** | 7.11E-02 | -0.39 | 3.39E-02 | -0.47 | ***9.69E-05*** | ***-1.03*** |
| TMEM132D | 1.28E-02 | -1.17 | 3.47E-02 | -0.97 | 3.76E-02 | -0.96 | ***1.18E-02*** | ***-1.19*** |
| TPD52 | 6.57E-01 | 0.12 | 1.52E-02 | -0.73 | ***4.75E-03*** | ***-0.82*** | 4.89E-01 | 0.21 |
| UBA52 | ***8.03E-04*** | ***-0.66*** | 7.10E-03 | -0.50 | 3.13E-03 | -0.56 | ***1.86E-03*** | ***-0.60*** |
| VGF | ***1.35E-03*** | ***-0.98*** | 1.87E-02 | -0.68 | 2.65E-02 | -0.63 | ***9.21E-04*** | ***-1.02*** |
| VSTM2A | ***3.00E-04*** | ***-1.03*** | 4.46E-02 | -0.51 | 1.92E-02 | -0.60 | ***7.75E-04*** | ***-0.93*** |
| VSTM2B | ***4.27E-05*** | ***-0.77*** | 1.55E-02 | -0.39 | 2.24E-03 | -0.52 | ***3.14E-04*** | ***-0.64*** |
| VTN | ***7.97E-04*** | ***0.77*** | ***1.73E-03*** | ***0.70*** | ***3.51E-03*** | ***0.64*** | ***3.88E-04*** | ***0.83*** |
