# Supplementary material for: Proteomics and mathematical modeling of longitudinal CSF differentiates fast versus slow ALS progression
Source: Ann Clin Transl Neurol. 2023 Aug 30;10(11):2025–42. doi: 10.1002/acn3.51890 (PMC10647001; doi:10.1002/acn3.51890)
Supplement: Supplementary file 3 — Figure S1 Caption [file ACN3-10-2025-s003.docx]

**Figure S1** StringDB analysis of the 59 candidate biomarkers. 47 proteins have known interactions with each other while 12 proteins; GFRA2, FCN3, AZGP1, AFM, SERPINA6, PGLYRP2, GPLD1, CAMK2A, CD300A, C1RL, ERAP1, FAM19A2 were not known to interact with any of the other candidate protein biomarkers.
